# [^225^Ac]Ac-SibuDAB for Targeted Alpha Therapy of Prostate Cancer: Preclinical Evaluation and Comparison with [^225^Ac]Ac-PSMA-617

**DOI:** 10.3390/cancers14225651

**Published:** 2022-11-17

**Authors:** Sarah D. Busslinger, Viviane J. Tschan, Olivia K. Richard, Zeynep Talip, Roger Schibli, Cristina Müller

**Affiliations:** 1 Center for Radiopharmaceutical Sciences ETH-PSI, Paul Scherrer Institute, Forschungsstrasse 111, 5232 Villigen-PSI, Switzerland; 2 AnaPath Services, Hammerstrasse 49, 4410 Liestal, Switzerland; 3 Department of Chemistry and Applied Biosciences, Vladimir-Prelog-Weg 1-5/10, ETH Zurich, 8093 Zurich, Switzerland

**Keywords:** actinium-225, PSMA, SibuDAB, PSMA-617, prostate cancer, alpha therapy, albumin binder

## Abstract

**Simple Summary:**

Prostate-specific membrane antigen (PSMA) radioligands have proven effective to treat patients with metastatic castration-resistant prostate cancer. Targeted α-therapy using actinium-225 has been used for patients with end-stage disease who no longer responded to β^ࢤ^-therapy through the use of ^177^Lu-based radioligand therapy (RLT). In this study, we investigated and compared the therapeutic efficacy of [^225^Ac]Ac-SibuDAB and [^225^Ac]Ac-PSMA-617 and assessed potential undesired side effects in the preclinical setting. Due to a dedicated albumin-binding entity integrated into [^225^Ac]Ac-SibuDAB, this radioligand showed an enhanced blood circulation time and, hence, increased tumor uptake but also higher retention in normal tissues. The therapeutic efficacy of [^225^Ac]Ac-SibuDAB was enhanced as compared to that of [^225^Ac]Ac-PSMA-617, yet, undesired side effects were in the same range for both radioligands. Our data suggest that [^225^Ac]Ac-SibuDAB could be a powerful alternative to [^225^Ac]Ac-PSMA-617, however, the safe therapeutic window should be carefully defined in clinical dose escalation studies.

**Abstract:**

In the present study, SibuDAB, an albumin-binding PSMA ligand, was investigated in combination with actinium-225 and the data were compared with those of [^225^Ac]Ac-PSMA-617. In vitro, [^225^Ac]Ac-SibuDAB and [^225^Ac]Ac-PSMA-617 showed similar tumor cell uptake and PSMA-binding affinities as their ^177^Lu-labeled counterparts. The in vitro binding to serum albumin in mouse and human blood plasma, respectively, was 2.8-fold and 1.4-fold increased for [^225^Ac]Ac-SibuDAB as compared to [^177^Lu]Lu-SibuDAB. In vivo, this characteristic was reflected by the longer retention of [^225^Ac]Ac-SibuDAB in the blood than previously seen for [^177^Lu]Lu-SibuDAB. Similar to [^225^Ac]Ac-PSMA-617, [^225^Ac]Ac-SibuDAB was well tolerated at 30 kBq per mouse. Differences in blood cell counts were observed between treated mice and untreated controls, but no major variations were observed between values obtained for [^225^Ac]Ac-SibuDAB and [^225^Ac]Ac-PSMA-617. [^225^Ac]Ac-SibuDAB was considerably more effective to treat PSMA-positive tumor xenografts than [^225^Ac]Ac-PSMA-617. Only 5 kBq per mouse were sufficient to eradicate the tumors, whereas tumor regrowth was observed for mice treated with 5 kBq [^225^Ac]Ac-PSMA-617 and only one out of six mice survived until the end of the study. The enhanced therapeutic efficacy of [^225^Ac]Ac-SibuDAB as compared to that of [^225^Ac]Ac-PSMA-617 and reasonable safety data qualify this novel radioligand as a candidate for targeted α-therapy of prostate cancer.

## 1. Introduction

Prostate-specific membrane antigen (PSMA)-targeted radioligand therapy (RLT) has emerged as an efficient treatment strategy for metastatic castration-resistant prostate cancer (mCRPC) [1]. Based on the positive outcome of the clinical Phase III trial (VISION; NCT03511664 [2]), [^177^Lu]Lu-PSMA-617 was recently approved by the U.S. food and drug administration (FDA) under the trade name Pluvicto^TM^ for the treatment of patients with mCRPC. Despite these overall positive data for ^177^Lu-based RLT, up to 30% of the patients have shown inherent or acquired resistance over time [3]. In these cases, targeted α-therapy may present an effective option, since α-particles are characterized by a short tissue range (≤100 µm) and a substantially higher linear energy transfer (LET; 50–300 keV/µm) [4].

RLT using [^225^Ac]Ac-PSMA-617 and [^225^Ac]Ac-PSMA-I&T was applied to patients that did not respond to β¯-therapy or suffered from multiple bone lesions, which presents a contraindication for ^177^Lu-based RLT due to the increased risk of hematologic toxicity [5,6]. Both, [^225^Ac]Ac-PSMA-617 and [^225^Ac]Ac-PSMA-I&T revealed to be effective in the clinical setting to treat end-stage prostate cancer [7]. A clinical Phase I dose escalation study to evaluate the safety of [^225^Ac]Ac-PSMA-617 (AcTION: NCT04597411) in prostate cancer patients with extensive skeletal metastases is currently ongoing. At the same time, a clinical Phase II study (TATCIST: NCT05219500) is in progress to investigate the therapeutic efficacy of [^225^Ac]Ac-PSMA-I&T using a de-escalation scheme.

^225^Ac-based RLT led to significant reduction of the tumor burden and biochemical response with >50% prostate-specific antigen (PSA) decline in the majority of mCRPC patients [7]. Bone marrow toxicity, damage to the salivary glands and, on a longer term, also to the kidneys was observed frequently in treated patients as a result of the high absorbed radiation dose in these organs and tissues [8,9]. In particular, irreversible xerostomia due to salivary gland damage significantly compromised the patients’ quality of life [7,10]. Lower activities applied at shorter intervals were, therefore, recommended to alleviate potential side effects regarding the salivary glands [8]. Furthermore, specific measures to reduce salivary gland uptake, such as the cooling during radioligand application or the injection of botulinum toxin prior to ^225^Ac-based RLT showed variable efficacy [11].

The development of innovative PSMA radioligands that show reduced salivary gland accumulation relative to the tumor uptake would certainly present the smartest solution to address the challenge of salivary gland toxicity. In this context, the question arises whether albumin-binding radioligands, which show enhanced blood retention and increased tumor accumulation, would be advantageous in this regard. So far, only few long-circulating PSMA ligands were investigated in combination with actinium-225 [12,13], but preclinical data are not predictive for salivary gland accumulation in patients. Initial clinical application of [^177^Lu]Lu-EB-PSMA-617, a radioligand modified with Evans Blue as an albumin-binding entity, showed a significantly increased salivary gland accumulation compared to that of [^177^Lu]Lu-PSMA-617 [14]. In contrast, a first-in-human application of [^177^Lu]Lu-PSMA-ALB-56, a PSMA radioligand modified with a *p*-tolyl-butanoyl entity as an albumin binder, revealed similar salivary gland accumulation as [^177^Lu]Lu-PSMA-617 and, therefore, a favorable tumor-to-salivary gland dose ratio [15].

Recently, we have developed a new class of PSMA ligands comprising ibuprofen as an albumin-binding entity [16], whereof SibuDAB emerged as the most promising candidate [17]. The calculation of the area under the curve (AUC) values revealed higher tumor uptake for [^177^Lu]Lu-SibuDAB than for [^177^Lu]Lu-PSMA-617 but reduced retention in blood and kidneys as compared to [^177^Lu]Lu-PSMA-ALB-56. Based on the tissue distribution profile of [^177^Lu]Lu-SibuDAB, the application of SibuDAB in combination with actinium-225 appeared, thus, feasible and reasonable to be investigated.

**Figure 1:**
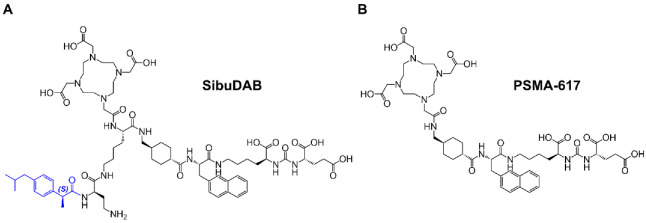
Chemical structures of the PSMA ligands. (**A**) SibuDAB, referring to a PSMA ligand equipped with the (*S*)-ibuprofen (blue) as an albumin-binding entity conjugated via diaminobutyric acid (DAB) and a lysine residue [17]; (**B**) PSMA-617 as a reference compound [18].

The aim of this study was to investigate ^225^Ac-based RLT using SibuDAB and compare the antitumor efficacy with that of [^225^Ac]Ac-PSMA-617 (Figure 1). In a first step, [^225^Ac]Ac-SibuDAB was evaluated in vitro for comparison with [^225^Ac]Ac-PSMA-617 and their ^177^Lu-labeled counterparts using PC-3 PIP (PSMA-positive) and PC-3 flu (PSMA-negative) prostate cancer cells. In a second step, the two ^225^Ac-based radioligands were compared with regard to potential early side effects in immunocompetent mice followed by investigations of their pharmacokinetic profiles and therapeutic efficacy in PC-3 PIP tumor-bearing mice.

## 2. Materials and Methods

### 2.1. Preparation of the Radioligands

The PSMA ligands used in this study were synthesized using solid-phase chemistry methods as previously reported [17,18]. The labeling of the PSMA ligands with actinium-225 was performed at ITM Medical Isotopes GmbH, Munich, Germany (Supplementary Material: Section S1). The radioligands (~50 kBq/nmol; ~0.5 MBq/mL) were shipped to the Paul Scherrer Institute (PSI) overnight followed by assessing their integrity using thin-layer chromatography. It was demonstrated that both radioligands, [^225^Ac]Ac-SibuDAB and [^225^Ac]Ac-PSMA-617, were stable over a period of 48 h (>99% intact radioligand) and could, therefore, be used for preclinical experiments on the two following days upon arrival. Depending on the experiment, the molar activity of the radioligands was adjusted by adding unlabeled ligand and the final formulation was prepared by adding vehicle (saline with 0.05% bovine serum albumin (BSA)) to obtain the desired activity concentration. Radiolabeling of SibuDAB with lutetium-177 (5–10 MBq/nmol) was performed as previously reported [17].

### 2.2. Activity Measurement of ^225^Ac-Samples

At PSI, the measurement of the activity was performed based on the γ-emission of bismuth-213 (Eγ = 440 keV; I = 25.94%) using a high-purity germanium detector (Canberra, France), in combination with the Inter-Winner software package (version 7.1, Itech Instruments, France). Relative counting of ^225^Ac-samples was performed using a γ-counter (Perkin Elmer, Wallac Wizard 1480) with a set energy window of 20–800 keV to detect the γ-emissions of francium-221 (Eγ = 218 keV; I = 11.44%) and bismuth-213 (Eγ = 440 keV; I = 25.94%) (Supplementary Material: Section S2).

### 2.3. Cell Culture

PSMA-positive PC-3 PIP and PSMA-negative PC-3 flu prostate cancer cells were kindly provided by Prof. Dr. Martin Pomper (Johns Hopkins University School of Medicine, Baltimore, MD, USA). These androgen-independent cancer cells were cultured in RPMI-1640 cell culture medium supplemented with 10% fetal calf serum, L-glutamine, antibiotics and puromycin (2 µg/mL) [19].

### 2.4. In Vitro Characterization

The logD values, cell uptake and internalization as well as PSMA-binding affinities (K_D_ values) of [^225^Ac]Ac-SibuDAB and [^225^Ac]Ac-PSMA-617 (~50 kBq/nmol) were determined according to previously reported protocols (Supplementary Material: Sections S3–S5) [16,17]. Relative albumin-binding affinities were determined using an ultrafiltration assay according to an adapted version of a recently published protocol (Supplementary Material: Section S6) [20].

### 2.5. In Vivo Experiments

All applicable international, national, and/or institutional guidelines for the care and use of laboratory animals were followed and all animal experiments were carried out according to the guidelines of Swiss Regulations for Animal Welfare. The preclinical studies were ethically approved by the Cantonal Committee of Animal Experimentation and permitted by the responsible cantonal authorities (license N° 75668). Female mice were used as they can be housed in groups over a long period without the risk of fighting with each other as it is commonly seen with male mice. This is relevant, in particular for studies performed over longer periods. Female mice were also chosen for the tumor therapy study, which is feasible when using an androgen-independent prostate cancer model.

### 2.6. Tolerability Study in Immunocompetent Mice

#### 2.6.1. Design of the Tolerability Study

Five- to six-week-old immunocompetent, female FVB mice were purchased from Charles River Laboratories (Sulzfeld, Germany). After an acclimatization period of at least 7 days, the mice (*n* = 5 per group) were intravenously injected with 30 kBq [^225^Ac]Ac-SibuDAB or 30 kBq [^225^Ac]Ac-PSMA-617 and additional mice (*n* = 7 per group) remained untreated as controls. Mice were monitored by measuring their body mass and potential signs of pain or unease three times a week (Supplementary Material: Section S7). The mice were euthanized on Day 10, Day 28 or on Day 56, respectively, and variable parameters were assessed from blood and tissue samples.

#### 2.6.2. Blood Sampling and Assessment of Parameters Indicative for Tolerability

Hematological analysis was performed using blood samples taken by cardiac puncture immediately after euthanasia. Blood and bone marrow smears were prepared and stained using the Pappenheim protocol [21]. The assessment was carried out by a board-certified veterinary anatomic pathologist (AnaPath Services GmbH, Liestal, Switzerland). Blood plasma parameters were determined in plasma after centrifugation of blood samples obtained shortly before euthanasia (Supplementary Material: Section S7).

#### 2.6.3. Histopathology

Tissue sections of formalin-fixed paraffin-embedded (FFPE) spleen, salivary glands and kidneys were prepared and stained with hematoxylin and eosin (H&E) by AnaPath Services GmbH (Liestal, Switzerland). The tissues were analyzed by a board certified veterinary pathologist using a pre-defined scoring system (Supplementary Material: Section S7).

### 2.7. Biodistribution Studies

Five- to six-week-old female BALB/c nude mice were purchased from Charles River Laboratories (Sulzfeld, Germany). Approximately 2 weeks after subcutaneous inoculation of PC-3 PIP and PC-3 flu tumor cells (6 × 10^6^ cells and 5 × 10^6^ cells, respectively, in 100 μL Hanks’ balanced salt solution (HBSS)) on the right and left shoulder, respectively, biodistribution studies were performed. [^225^Ac]Ac-SibuDAB and [^225^Ac]Ac-PSMA-617 (40 kBq; 1 nmol, 100 µL saline containing 0.05% BSA per mouse) were intravenously injected into *n* = 4–5 mice per timepoint. Collected tissues and organs were weighed and counted for activity using a γ-counter (PerkinElmer Wallac Wizard 1480) the day after dissection. Biodistribution data were reported as the percentage of the injected activity per gram of tissue mass (% IA/g) obtained from decay-corrected data (Supplementary Material: Section S8).

### 2.8. Therapy Studis in Tumor-Bearing Mice

#### 2.8.1. PSMA-Specific Antitumor Efficacy of [^225^Ac]Ac-SibuDAB

Five days after subcutaneous inoculation of four groups of mice with either PC-3 PIP or PC-3 flu tumor cells on the right shoulder (4 × 10^6^ cells in 100 μL HBSS), the mice were intravenously injected with either vehicle only (saline with 0.05% BSA, *n* = 6 and *n* = 4, respectively) or with 30 kBq [^225^Ac]Ac-SibuDAB (1 nmol per mouse; *n* = 4 per group) diluted in vehicle (Table 1).

#### 2.8.2. Therapeutic Efficacy of [^225^Ac]Ac-SibuDAB and [^225^Ac]Ac-PSMA-617

After tumor cell inoculation, mice bearing PC-3 PIP tumor xenografts were intravenously injected with vehicle only (*n* = 6) or with [^225^Ac]Ac-SibuDAB or [^225^Ac]Ac-PSMA-617, respectively, applied at 5 kBq or 10 kBq (1 nmol per mouse; *n* = 6 per group; Table 1). The control group consisted of mice from several studies (*n* = 12 mice) and the data from this group were published previously [17].

#### 2.8.3. Monitoring of Treated Mice and Therapy Assessment

After administration of the radioligands, the mice were monitored over 8 weeks using the same measures as previously published by Umbricht et al. [22]. The relative body mass was defined as [BM_x_/BM_0_], where BM_x_ is the body mass in grams at a given Day x and BM_0_ is the body mass in grams at Day 0. The tumor dimension was determined by measuring the longest tumor axis (L) and its perpendicular axis (W) with a digital caliper. The tumor volume (V) was calculated according to the equation [V = 0.5 × (LW^2^)]. The relative tumor volume was defined as [TV_x_/TV_0_], where TV_x_ is the tumor volume in mm^3^ at a given Day x, and TV_0_ is the tumor volume in mm^3^ at Day 0. Endpoint criteria that required euthanasia were defined as (i) body mass loss of >15%, (ii) a tumor volume of >800 mm^3^, (iii) a combination of body mass loss of >10% and a tumor volume of >700 mm^3^ or, (iv) signs of unease and pain or a combination thereof using a scoring system as previously reported [22]. The median survival of mice of each group was assessed with Kaplan–Meier curves. Potential early side effects for mice that survived until study end were determined based on blood chemistry and organ-to-brain mass ratios on Day 56 by comparison to values obtained from an additional group of therapy-naïve mice (*n* = 4) without tumors but of the same age (Supplementary Material: Section S9).

### 2.9. Analysis of Statistical Significance of the Data

The data of the in vitro and in vivo studies were analyzed using GraphPad Prism software (version 8) with a *p*-value < 0.05 considered as statistically significant. The cell uptake and internalization studies, the biodistribution studies, the body mass and tumor volume of mice on Day 0 of the therapy study, as well as parameters to determine adverse events during the tolerability and therapy studies were analyzed and compared using a one-way ANOVA test with a Tukey’s multiple comparisons post-test. The efficacy of the therapy study was assessed using a log-rank test (Mantel Cox).

## 3. Results and Discussion

### 3.1. In Vitro Characteristics of the ^225^Ac- and ^177^Lu-Based PSMA Radioligands

In a first step, the aim was to investigate the in vitro features of [^225^Ac]Ac-SibuDAB and to compare them with those of [^225^Ac]Ac-PSMA-617. Furthermore, the question whether actinium-225 and lutetium-177 would be interchangeable without affecting the radioligands’ physicochemical properties was addressed.

#### 3.1.1. Distribution Coefficients of ^225^Ac- and ^177^Lu-Based PSMA Radioligands

The logD value of [^225^Ac]Ac-SibuDAB (−2.5 ± 0.1) was in the same range as for the ^177^Lu-labeled analogue (−2.3 ± 0.1) [17], and the same was likely the case for the logD values of [^225^Ac]Ac-PSMA-617 (≤−3.9) and [^177^Lu]Lu-PSMA-617 (−4.4 ± 0.2) [23], although an exact determination was not possible for [^225^Ac]Ac-PSMA-617 due to the detection limit for the measurement of actinium-225. These data suggest that actinium-225 and lutetium-177 are interchangeable without significantly affecting the radioligands’ hydrophilicity. Due to the modification with ibuprofen, which increased the lipophilicity of radiolabeled SibuDAB, its logD value was higher than that of radiolabeled PSMA-617.

#### 3.1.2. Cell Uptake and PSMA-Binding of ^225^Ac- and ^177^Lu-Based PSMA Radioligands

**Figure 2:**
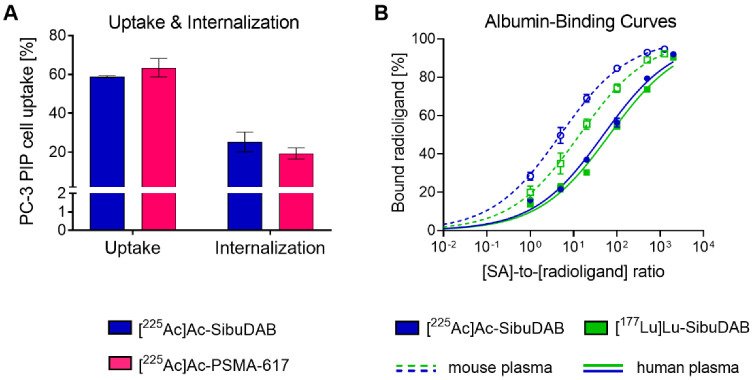
(**A**) Cell uptake and internalization of the ^225^Ac-labeled PSMA ligands into PSMA-positive PC-3 PIP cells (average ± SD, *n* = 3–4). (**B**) Comparison of albumin-binding curves of [^225^Ac]Ac-SibuDAB and [^177^Lu]Lu-SibuDAB determined in mouse and human blood plasma. The blood plasma was diluted to obtain a series of different molar concentration ratios of serum albumin (SA) to radioligand.

Cell uptake and internalization of [^225^Ac]Ac-SibuDAB (59 ± 1% and 25 ± 5%, respectively) into PC-3 PIP tumor cells was in the same range as for [^225^Ac]Ac-PSMA-617 (63 ± 5% and 19 ± 3%, respectively) and their respective ^177^Lu-labeled counterparts [17,24] after a 4 h-incubation period (*p* > 0.05) (Figure 2A, Figure S1). PSMA-mediated uptake was confirmed by the fact that the uptake of both radioligands into PSMA-negative PC-3 flu cells was <1%.

PSMA-binding affinities of [^225^Ac]Ac-SibuDAB (K_D_ value: 17 nM; CI: 11–23 nM) and [^225^Ac]Ac-PSMA-617 (K_D_ value: 11 nM; CI: 6–16 nM), respectively, were in the same range and similar to previously published data obtained with the ^177^Lu-labeled analogues (Table S1) [16,17]. These in vitro studies confirmed that actinium-225 and lutetium-177 are interchangeable without significantly affecting the radioligands’ PSMA-binding affinity nor uptake and internalization into PSMA-positive PC-3 PIP cells similarly as it was recently shown with ^177^Lu- and ^225^Ac-labeled PSMA-I&T [25].

#### 3.1.3. Serum Albumin Binding of [^225^Ac]Ac-SibuDAB and [^177^Lu]Lu-SibuDAB

The relative albumin-binding affinity of [^225^Ac]Ac-SibuDAB determined in mouse and human blood plasma, respectively, was approximately 2.8-fold and 1.4-fold higher than for [^177^Lu]Lu-SibuDAB (Figure 2B). Possibly, the larger radius of actinium-225 changed the conformation of the molecule and, hence, the binding mode to serum albumin. The fact that this effect was much more pronounced in mouse than in human plasma lessens, however, its relevance in view of an application of [^225^Ac]Ac-SibuDAB in human patients. It is also important to note that both [^225^Ac]Ac-SibuDAB and [^177^Lu]Lu-SibuDAB revealed a stronger binding to mouse serum albumin than to human serum albumin, hence, pharmacokinetic data acquired in mice may not precisely reflect the situation in humans as to what concerns the radioligands’ blood circulation time.

### 3.2. Tolerability of [^225^Ac]Ac-SibuDAB in Immunocompetent Mice

Common undesired side effects of RLT with [^225^Ac]Ac-PSMA-617 in clinics include hematological changes, xerostomia and impairment of kidney function [26]. Hematotoxicity up to Grade 3/4 was observed in about 30% of the patients in a clinical study reported by Feuerecker et al. [8]. Xerostomia was sometimes associated with reduced appetite and body mass loss, and a decreased quality of life has been reported in the majority of patients treated with [^225^Ac]Ac-PSMA-617 [26]. The topic of kidney radiotoxicity is controversially discussed among clinicians in the community. A clear conclusion about how ^225^Ac-based RLT affects long-term kidney function is still difficult to draw as [^225^Ac]Ac-PSMA-617 has been used for the treatment of end-stage prostate cancer patients with a limited life expectancy.

In this preclinical study, we investigated the tolerability of [^225^Ac]Ac-SibuDAB and [^225^Ac]Ac-PSMA-617 in immunocompetent mice. According to recently reported preclinical studies [12,27,28], an activity of 20–40 kBq [^225^Ac]Ac-PSMA-617 was effective to control tumor growth without causing major side effects. [^225^Ac]Ac-SibuDAB and [^225^Ac]Ac-PSMA-617 were, therefore, applied at 30 kBq per mouse. Indeed, both radioligands were well tolerated based on the average body mass of mice of each group, which increased similarly over time until the end of the study on Day 56 (Figure S2).

#### 3.2.1. Hematological Effects of [^225^Ac]Ac-SibuDAB and [^177^Lu]Lu-SibuDAB

The complex decay chain of actinium-225 with several α-particle-emitting daughter nuclides could potentially present a risk of side effects [29]. In this regard, the efficient internalization of PSMA upon binding of PSMA radioligands is certainly an advantage, whereas the enhanced circulation time of [^225^Ac]Ac-SibuDAB may increase the number of decays outside the target cell. Furthermore, a longer blood circulation time would certainly increase the risk of bone marrow toxicity. Most likely, the applicable activity of [^225^Ac]Ac-SibuDAB would, therefore, have to be adjusted.

**Figure 3:**
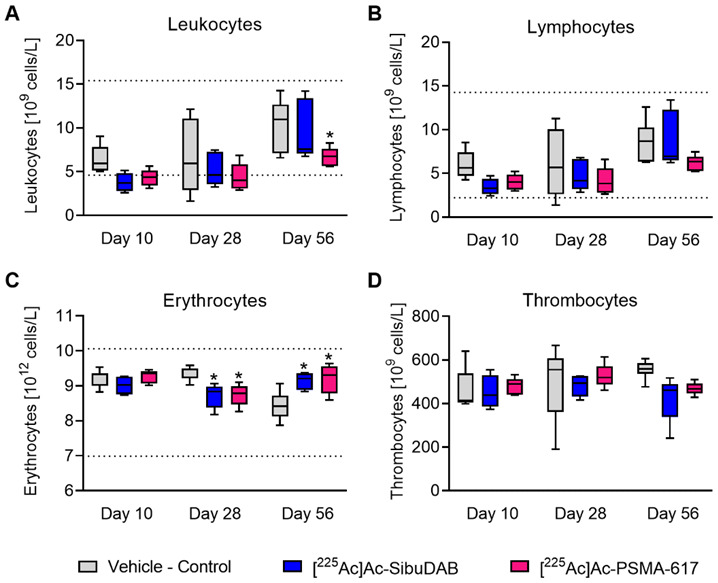
Hematological analysis of the blood from mice (*n* = 4–7) at defined days after treatment with vehicle or 30 kBq of either PSMA radioligand. (**A**) Leukocyte counts; (**B**) Lymphocyte counts; (**C**) Erythrocyte counts; (**D**) Thrombocyte counts. Values that were significantly different from those of control mice were indicated with an asterisk (*). The dotted lines represent reference values for healthy female FVB mice previously reported by Schneck et al. [30].

The hemograms of mice treated with [^225^Ac]Ac-SibuDAB or [^225^Ac]Ac-PSMA-617 did not differ significantly at any of the investigated timepoints (*p* > 0.05). Differences in hematology were, however, observed between treated mice and controls, although most values were still in the normal range based on the values reported for female FVB mice (Figure 3; Figure S3) [30]. A trend of lower leukocyte counts due to a drop in lymphocyte counts was observed on Day 10 and Day 28 in treated mice as compared to control mice (*p* > 0.05; Figure 3A,B). On Day 56, the difference was only statistically significant for mice that received [^225^Ac]Ac-PSMA-617 (*p* < 0.05) but not for those that received [^225^Ac]Ac-SibuDAB (*p* > 0.05). Erythrocyte counts declined to (8.7 ± 0.3) × 10^12^/L on Day 28 after administration of either radioligand as compared to control mice ((9.3 ± 0.2) × 10^12^/L; *p* < 0.05) (Figure 3C). In line with this, the hematocrit and hemoglobin levels also decreased on Day 28 after application of [^225^Ac]Ac-SibuDAB (43 ± 1% and 11.2 ± 0.5 g/dL, respectively) and [^225^Ac]Ac-PSMA-617 (43 ± 3% and 11.3 ± 0.5 g/dL, respectively) as compared to those of untreated controls (46 ± 2% and 12.1 ± 0.5 g/dL, respectively; *p* < 0.05; Figure S3). As a result of compensatory mechanisms, the erythrocyte counts, hematocrit and hemoglobin showed higher values than control mice on Day 56. No significant differences between thrombocyte counts of treated and untreated mice were observed at any of the investigated timepoints (Figure 3D).

To compensate for the decreased number of leukocytes determined on Day 10, the majority of mice (three out of five in each group) that had received an ^225^Ac-based RLT showed an increase in myleopoietic precursor cells in relation to erythropoietic precursor cells in the bone marrow smears as compared to control mice at that timepoint (Table S2–S4). On Day 28, the ratio tended to be shifted towards the erythorpoietic precursor cells to compensate for the lower number of erythrocytes determined in peripheral blood at that timepoint. No morphological cell abnormalities were observed in blood and bone marrow smears which would indicate severe hematological toxicity.

Histopathological analysis of the spleen, an organ involved in hematopoiesis, revealed a minimal to mild hyperplasia visible on Day 28 in ^225^Ac-treated mice but showed otherwise no major differences between mice that received ^225^Ac-based RLT and mice of the control group (Figure S4, Tables S5 and S6).

Under the given experimental conditions, hematological differences between treated and untreated mice were observed, however, it can be concluded that no signs of an increased risk of hematological toxicity were identified for the use of [^225^Ac]Ac-SibuDAB as compared to [^225^Ac]Ac-PSMA-617.

#### 3.2.2. Transient Salivary Gland Lesions after Application of ^225^Ac-Based Radioligands

The high salivary gland accumulation of [^225^Ac]Ac-PSMA-617 in humans can cause irreversible Grade 1/2 xerostomia in patients already after the first therapy cycle [8,31]. It is still unclear whether albumin-binding radioligands would show less or more salivary gland uptake in patients. While albumin-binding PSMA radioligands showed consistently increased tumor uptake in patients as compared to conventional radioligands, Zang et al. reported on concomitantly increased salivary gland accumulation of [^177^Lu]Lu-EB-PSMA-617 (6.41 ± 1.40 Gy/GBq) [14], whereas Kramer et al. found comparable salivary gland uptake of [^177^Lu]Lu-PSMA-ALB-56 (0.86 ± 0.42 Gy/GBq) [15] as was reported for [^177^Lu]Lu-PSMA-617 (1.41 ± 0.53 Gy/GBq) [32]. A clear conclusion about whether the use of albumin-binding PSMA radioligands would be favorable remains to be elucidated in future clinical studies.

**Figure 4:**
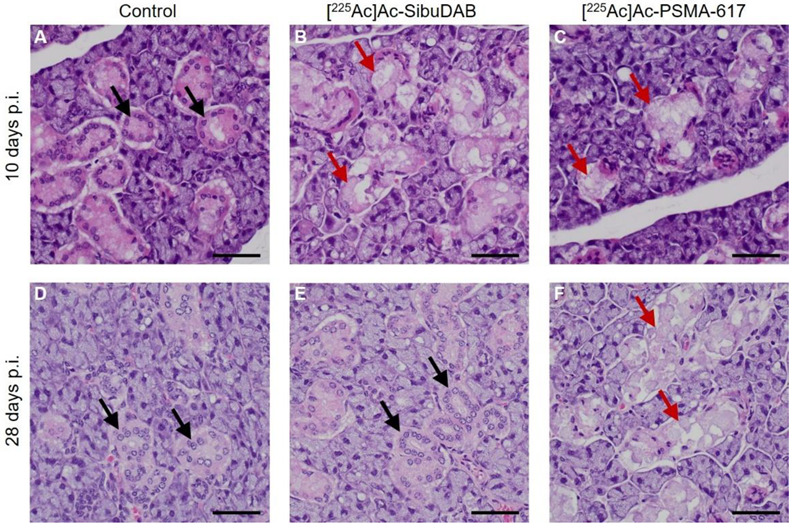
Representative images of H&E stained FFPE histological sections of the salivary glands on Day 10 and Day 28 of (**A**,**D**) control mice, (**B**,**E**) mice injected with [^225^Ac]Ac-SibuDAB and (**C**,**F**) mice injected with [^225^Ac]Ac-PSMA-617. The red arrows highlight moderate necrosis of the serous acinar cells; black arrows show intact serous acinar cells. The scale bar corresponds to a length of 100 μm.

Histopathological analysis of the sublingual salivary glands revealed minimal to moderate necrosis of the serous acinar cells, characterized by karyorrhexis and pyknosis with loss of cellular architecture, on Day 10 after application of [^225^Ac]Ac-SibuDAB and [^225^Ac]Ac-PSMA-617 in two out of five mice, each. On Day 28, these lesions were observed in all five mice injected with [^225^Ac]Ac-PSMA-617, but completely absent in mice treated with [^225^Ac]Ac-SibuDAB (Figure 4, Tables S5 and S6). On Day 56, none of the mice showed these lesions, indicating that also the salivary glands of mice that received [^225^Ac]Ac-PSMA-617 fully recovered. The observed changes in the salivary glands were transient and more pronounced after injection of [^225^Ac]Ac-PSMA-617 than after application of [^225^Ac]Ac-SibuDAB. A possibly similar observation was made by Banerjee et al. who reported on smaller acini and higher cytoplasmic variation in salivary glands of mice one year after the treatment with [^225^Ac]Ac-L1 (2 × 18.5 kBq), a recently developed radioligand [33].

The data of our study may indicate that albumin-binding radioligands are advantageous in view of salivary gland toxicity even though a prediction for human application is not possible. The salivary gland cells and their organization are quite different in mice and humans [34]. This is probably the reason why radioligand uptake in humans is significantly higher as compared to the salivary gland uptake in mice, where it was mostly negligible [17].

#### 3.2.3. Assessment of Potential Kidney Damage after Application of [^225^Ac]Ac-SibuDAB

The risk of kidney toxicity after ^225^Ac-based RLT is controversially discussed among physicians and contradictive statements can be found in the literature. Feuerecker et al. [35] reported Grade 1/2 impairment of kidney function in about 19% of the patients of the respective study 4–8 weeks after treatment, however, similarly to previously published data [8], it was not of clinical relevance. Pelletier et al. reported two cases of progressive kidney disease in mCRPC patients with impaired baseline kidney function that received treatment with [^225^Ac]Ac-PSMA-617 [9]. A kidney biopsy performed for one of the patients 34 weeks after first exposure showed significant tubular atrophy and ongoing tubular injury.

In our preclinical study, we observed similar (*p* > 0.05) blood urea nitrogen levels in mice treated with [^225^Ac]Ac-SibuDAB (9.1 ± 1.1 mmol/L) and [^225^Ac]Ac-PSMA-617 (8.7 ± 1.1 mmol/L) on Day 28. In the case of [^225^Ac]Ac-SibuDAB these values were significantly elevated as compared to those of control mice (6.7 ± 0.7 mmol/L, *p* < 0.05), however, this was no longer the case on Day 56 (Figure S5). Histopathological evaluation did not reveal any changes other than background lesions for the kidneys of treated mice and untreated controls (Tables S5 and S6). An increased risk of acute nephrotoxicity after application of [^225^Ac]Ac-SibuDAB as compared to [^225^Ac]Ac-PSMA-617 was not identified under the given experimental conditions. It has to be critically acknowledged, however, that radionephropathy in patients is commonly observed later than two months after administration of the radioligand [9,36]. Although the manifestations may occur earlier in mice than in humans, delayed radiotoxic effects cannot be excluded based on the data presented in this study. In an ideal case, the observation period of mice should be much longer to detect potential kidney toxicity as it was done by Banerjee et al. who assessed side effects of [^225^Ac]Ac-L1 one year after application [33]. It is obvious that, based on the increased kidney retention of [^225^Ac]Ac-SibuDAB, the injected activity would have to be reduced as compared to that of [^225^Ac]Ac-PSMA-617 in order not to increase the risk of radionephrotoxicity.

### 3.3. Tissue Distribution Profiles of [^225^Ac]Ac-SibuDAB and [^225^Ac]Ac-PSMA-617

In a next step, we investigated the tissue distribution profile of the radioligands using PC-3 PIP tumor-bearing mice in view of a therapeutic application of the radioligands that should allow an estimation of their antitumor efficacy.

#### 3.3.1. Biodistribution of [^225^Ac]Ac-SibuDAB and [^225^Ac]Ac-PSMA-617

**Figure 5:**
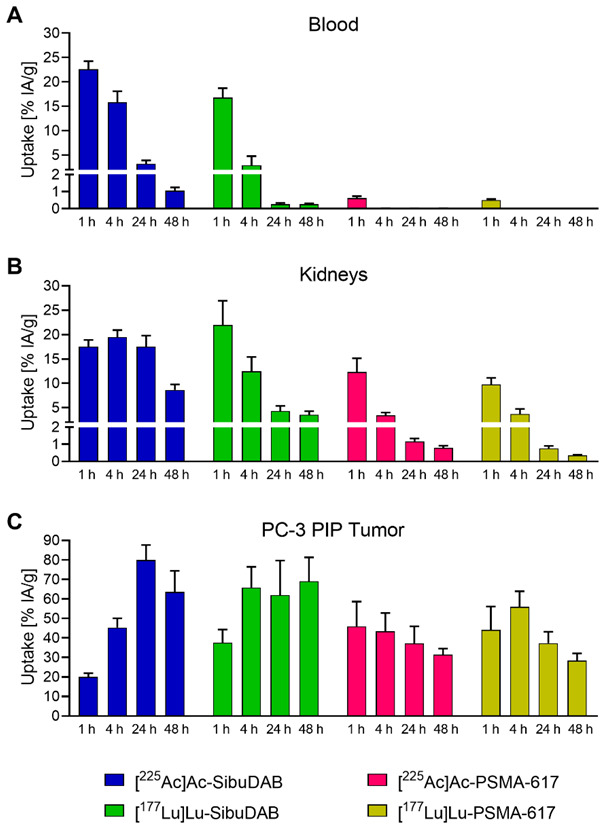
Accumulation of SibuDAB and PSMA-617 labeled with either actinium-225 or lutetium-177 in (**A**) blood, (**B**) kidneys and (**C**) PSMA-positive PC-3 PIP tumor xenografts. Data obtained for [^177^Lu]Lu-SibuDAB were previously published by Borgna & Deberle et al. Mol Pharm 2022 [17] and data of [^177^Lu]Lu-PSMA-617 were previously published by Benešová et al. Mol Pharm 2018 [23] (Copyrights 2022 and 2018, American Chemical Society) and were added for comparison.

Biodistribution data obtained at 1 h, 4 h, 24 h and 48 h after injection of the radioligands showed the expected differences between [^225^Ac]Ac-SibuDAB and [^225^Ac]Ac-PSMA-617 that were previously found with the ^177^Lu-labeled counterparts (Figure 5; Tables S7–S10). Blood retention of [^225^Ac]Ac-SibuDAB (23 ± 2% IA/g and 3.3 ± 0.7% IA/g at 1 h and 24 h post injection (p.i.), respectively) was enhanced as compared to that of [^225^Ac]Ac-PSMA-617 and the retention of [^225^Ac]Ac-SibuDAB in the kidneys was also higher (19 ± 1 % IA/g vs. 3.5 ± 0.6 % IA/g at 4 h p.i.). The tumor accumulation of [^225^Ac]Ac-SibuDAB was significantly higher than for [^225^Ac]Ac-PSMA-617 with a maximum value reached at 24 h p.i. (80 ± 8% IA/g). At 48 h p.i., a 2-fold higher tumor uptake was observed for [^225^Ac]Ac-SibuDAB as compared to [^225^Ac]Ac-PSMA-617 (64 ± 11% IA/g vs. 31 ± 3% IA/g). Unspecific accumulation in PSMA-negative PC-3 flu tumors was not observed.

#### 3.3.2. Tissue Distribution Profiles of [^225^Ac]Ac-SibuDAB and [^177^Lu]Lu-SibuDAB

Other than in the case of [^225^Ac]Ac-PSMA-617 and [^177^Lu]Lu-PSMA-617, the albumin-binding [^225^Ac]Ac-SibuDAB and [^177^Lu]Lu-SibuDAB revealed considerable differences in their tissue distribution profiles (Figure 5; Tables S7–S10). In line with the in vitro findings of an enhanced plasma protein–binding affinity of [^225^Ac]Ac-SibuDAB as compared to [^177^Lu]Lu-SibuDAB, the former showed an increased blood retention (16 ± 2% IA/g vs. 2.9 ± 1.9% IA/g at 4 h p.i.; Figure 5A). The altered clearance profile could potentially explain the observed differences in the renal uptake of [^225^Ac]Ac- and [^177^Lu]Lu-SibuDAB (Figure 5B). Differences in the tumor uptake were also visible at early timepoints after injection of [^225^Ac]Ac-SibuDAB as compared to the ^177^Lu-labeled counterpart (45 ± 5% IA/g vs. 66 ± 11% IA/g at 4 h p.i.; Figure 5C). Nevertheless, the activity retention in the tumor was similar at later timepoints (64 ± 11% IA/g vs. 69 ± 12% IA/g at 48 h p.i.). It has to be noted that the distribution profiles of [^225^Ac]Ac-SibuDAB and [^177^Lu]Lu-SibuDAB are likely more similar in humans because the albumin-binding affinities of the two radioligands were found to be more similar in human blood plasma than in mouse blood plasma.

### 3.4. Comparison of the Therapeutic Efficacy of ^225^Ac-Based Radioligands

#### 3.4.1. PSMA-Specific Treatment Effect of [^225^Ac]Ac-SibuDAB

**Figure 6:**
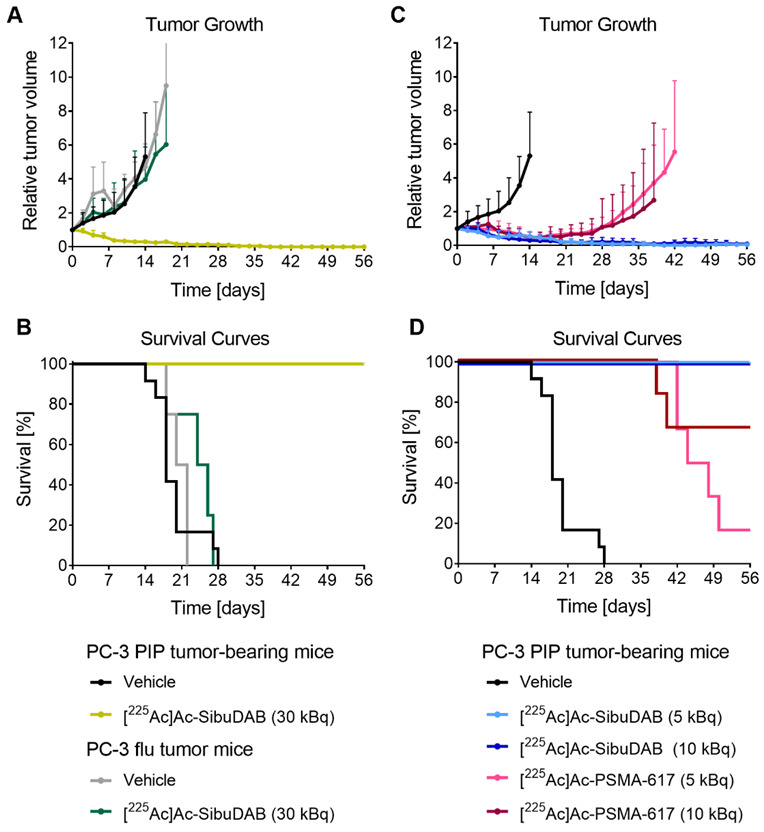
(**A**,**B**) PSMA-specific treatment efficacy of [^225^Ac]Ac-SibuDAB assessed in mice with PSMA-positive PC-3 PIP or PSMA-negative PC-3 flu tumors injected with either vehicle or 30 kBq radioligand. (**C**,**D**) Comparison of the therapeutic efficacy of [^225^Ac]Ac-SibuDAB and [^225^Ac]Ac-PSMA-617 (5 or 10 kBq) in PSMA-positive PC-3 PIP tumor-bearing mice. (**A**,**C**) Relative tumor growth shown until the first mouse of the respective group reached an endpoint; (**B**,**D**) Kaplan–Meier plots showing the survival curves of mice of each group. The data of the PC-3 PIP tumor-bearing control mice were previously published by Borgna & Deberle et al. Mol Pharm 2022 [17] (Copyright 2022, American Chemical Society).

PC-3 PIP and PC-3 flu tumor xenografts of untreated control mice rapidly increased in size so that all mice of these groups reached an endpoint between Day 14 and Day 28 or between Day 18 and Day 22, respectively, with median survival times of 18 and 21 days, respectively (Figure 6, Table 2). Application of 30 kBq [^225^Ac]Ac-SibuDAB eradicated PSMA-positive PC-3 PIP tumors entirely so that all mice of this group survived until the end of the study. Application of the same activity of [^225^Ac]Ac-SibuDAB (30 kBq/mouse) did, however, not significantly delay the growth of PSMA-negative PC-3 flu tumors resulting in a survival time that was not much longer than in untreated control mice (*p* > 0.05, median survival of 25 days) (Figure 6A,B, Table 2). This unambiguously confirmed that the therapeutic efficacy of [^225^Ac]Ac-SibuDAB was dependent on PSMA expression and not due to an unspecific effect of actinium-225 or its daughter nuclides. These data further suggested that possibly even lower activities would be sufficient to obtain tumor control with the albumin-binding radioligand.

#### 3.4.2. Comparison of the Efficacy of [^225^Ac]Ac-SibuDAB and [^225^Ac]Ac-PSMA-617

The treatment of mice with 5 kBq or 10 kBq [^225^Ac]Ac-SibuDAB effectively eradicated tumors of all mice of these groups, which survived until the end of the study on Day 56 (Figure 6C,D, Table 2).

On the other hand, the application of 10 kBq [^225^Ac]Ac-PSMA-617 per mouse resulted in the survival of four out of six mice until the end of the study. Application of 5 kBq [^225^Ac]Ac-PSMA-617 was even less effective. After initial regression, tumor re-growth was observed and, consequently, five out of six mice of this group reached an endpoint within the 56 days of investigation. Nevertheless, even this group showed still a significantly prolonged survival (*p* < 0.05) with a median survival of 46 days as compared to control mice. Other research groups used commonly higher activities of [^225^Ac]Ac-PSMA-617 than 10 kBq per mouse, however, in most of these cases LNCaP and C4-2 tumors were used, which express PSMA naturally, and, thus, at much lower expression levels than it is the case for the transduced PC-3 PIP tumors [12,37,38]. Similar therapeutic efficacy was, however, reported by Banerjee et al. who used the PC-3 PIP tumor mouse model for the investigation of the therapeutic efficacy of [^225^Ac]Ac-L1 [33].

The body mass of mice that were effectively treated with [^225^Ac]Ac-SibuDAB was increasing over time, indicating that the therapy was well tolerated (Figure S6). In order to further assess the therapy outcome, we compared several parameters of mice, which had a tumor initially but were cured after the treatment with [^225^Ac]Ac-SibuDAB, with treatment-naïve, non-tumor-bearing BALB/c nude mice of the same age. No obvious adverse events were observed in cured mice at Day 56 after injection of 30 kBq [^225^Ac]Ac-SibuDAB as compared to healthy mice (Tables S11–S13). The only exception was a lower kidney-to-brain mass ratio (0.62–0.68) determined in cured mice as compared to healthy mice (0.73 ± 0.04) which could potentially be a sign of a radiation induced impairment of the kidneys in mice that received [^225^Ac]Ac-SibuDAB.

#### 3.4.3. Comparison of ^225^Ac-Based RLT and ^177^Lu-Based RLT

For the treatment of patients, typically a ~1000-fold lower activity has been applied for [^225^Ac]Ac-PSMA-617 (~8 MBq per cycle) than for [^177^Lu]Lu-PSMA-617 (~7–9 GBq per cycle). Recent dosimetry estimations performed by Hwan Lee revealed, that under such circumstances, the equivalent radiation dose deposited by [^225^Ac]Ac-PSMA-617 in macrometastatic disease would be higher compared to [^177^Lu]Lu-PSMA-617 and even considerably higher in microscopic disease [39]. Our study confirmed this observation by demonstrating a better effect using 5 kBq or 10 kBq [^225^Ac]Ac-SibuDAB than a 1000-fold higher activity of [^177^Lu]Lu-SibuDAB (5 MBq and 10 MBq per mouse) (Figure S7). Only in two out of six mice treated with 5 MBq [^177^Lu]Lu-SibuDAB and in five out of six mice treated with 10 MBq [^177^Lu]Lu-SibuDAB the tumor xenografts disappeared completely without relapse until Day 56. Tumors of the other mice of these groups started to regrow after ~4 and ~7 weeks, respectively. In comparison, no tumors were visible at Day 56 after injection of 5 kBq [^225^Ac]Ac-SibuDAB.

## 4. Conclusions

The increased therapeutic efficacy of [^225^Ac]Ac-SibuDAB as compared to the clinically investigated [^225^Ac]Ac-PSMA-617 indicated an advantage of this novel albumin-binding PSMA radioligand for targeted α-therapy. Despite the differences regarding the biodistribution profile and the longer blood circulation time of [^225^Ac]Ac-SibuDAB as compared to [^225^Ac]Ac-PSMA-617, the preliminary data regarding tolerability in immunocompetent mice did not show an obvious increased risk when using [^225^Ac]Ac-SibuDAB instead of [^225^Ac]Ac-PSMA-617. Nevertheless, the safe therapeutic window for the application of [^225^Ac]Ac-SibuDAB should be defined in a carefully designed clinical dose-escalation study.

## 5. Patents

A patent application on PSMA ligands with ibuprofen as an albumin-binding entity has been filed by ITM Medical Isotopes SE, Munich, Germany.

## Figures and Tables

**Table 1:** Design of the therapy study including information about the number (*n*) of mice per group, the tumor type, the treatment, the injected activity and the average ± standard deviation (SD) of the tumor volume and body mass of mice of each group on Day 0.

Investigation of PSMA-Specific vs. Unspecific Effects of [^225^Ac]Ac-SibuDAB					
Tumor	Treatment	Injected Activity [kBq]	Tumor Volume ^1^ [mm^3^]	Body Mass ^1^ [g]	*n*
PC-3 PIP	Vehicle ^2^	-	69 ± 34	17.9 ± 1.1	12
PC-3 PIP	[^225^Ac]Ac-SibuDAB	30	84 ± 13	17.2 ± 1.2	4
PC-3 flu	Vehicle	-	45 ± 28	18.9 ± 1.3	4
PC-3 flu	[^225^Ac]Ac-SibuDAB	30	58 ± 11	18.3 ± 0.9	4
**Comparison of the Therapeutic Efficacy of [^225^Ac]Ac-SibuDAB and [^225^Ac]Ac-PSMA-617**					
Tumor	Treatment	Injected Activity [kBq]	Tumor Volume ^1^ [mm^3^]	Body Mass ^1^ [g]	*n*
PC-3 PIP	[^225^Ac]Ac-SibuDAB	5	76 ± 25	17.5 ± 1.0	6
PC-3 PIP	[^225^Ac]Ac-SibuDAB	10	65 ± 22	17.7 ± 0.9	6
PC-3 PIP	[^225^Ac]Ac-PSMA-617	5	72 ± 24	18.2 ± 1.2	6
PC-3 PIP	[^225^Ac]Ac-PSMA-617	10	56 ± 18	17.3 ± 0.7	6

^1^ No significant differences were determined between the values measured for each group (*p* > 0.05). ^2^ The control group consisted of mice from several studies (*n* = 12 mice) and the data from this group were published previously by Borgna & Deberle et al. Mol Pharm 2022 [17] (Copyright 2022, American Chemical Society).

**Table 2:** Parameters indicative for treatment efficacy, including the tumor type, the day the first and last mouse of each group was euthanized, median survival and number of mice that survived until Day 56.

Investigation of PSMA-Specific vs. Unspecific Effects of [^225^Ac]Ac-SibuDAB					
Tumor	Treatment	Activity [kBq]	Days of First and Last Endpoint	Median Survival	Number of Mice Alive on Day 56
PC-3 PIP	Vehicle ^1^	-	14; 28	18	0/12
PC-3 PIP	[^225^Ac]Ac-SibuDAB	30	56 ^2^	>56 ^3^	4/4
PC-3 flu	Vehicle	-	18; 22	21	0/4
PC-3 flu	[^225^Ac]Ac-SibuDAB	30	18; 27	25	0/4
**Comparison of the Therapeutic Efficacy of [^225^Ac]Ac-SibuDAB and [^225^Ac]Ac-PSMA-617**					
Tumor	Treatment	Activity [kBq]	Days of First and Last Endpoint	Median Survival	Number of Mice Alive on Day 56
PC-3 PIP	[^225^Ac]Ac-SibuDAB	5	56 ^2^	>56 ^3^	6/6
PC-3 PIP	[^225^Ac]Ac-SibuDAB	10	56 ^2^	>56 ^3^	6/6
PC-3 PIP	[^225^Ac]Ac-PSMA-617	5	42; 56 ^2^	46	1/6
PC-3 PIP	[^225^Ac]Ac-PSMA-617	10	38; 56 ^2^	>56 ^3^	4/6

^1^ Data of the control group were previously published by Borgna & Deberle et al. Mol Pharm 2022 [17] (Copyright 2022, American Chemical Society). ^2^ All mice were euthanized at the end of the study on Day 56, even if not all mice had reached an endpoint. ^3^ The exact median survival values could not be defined, since more than half of the mice survived until study end.

## Data Availability

The data presented in this study are available on request from the corresponding author.

## Supplementary Materials

The following supporting information can be downloaded at: https://www.mdpi.com/article/10.3390/cancers14225651/s1, Figure S1: Cell uptake and internalization of radiolabeled SibuDAB and PSMA-617; Figure S2: Relative body mass of non-tumor-bearing FVB mice; Figure S3: Analysis of hemoglobin and hematocrit after treatment with [^225^Ac]Ac-SibuDAB or [^225^Ac]Ac-PSMA-617. Figure S4: Representative images of H&E stained paraffin-embedded sections of the spleen; Figure S5: Blood plasma chemistry of mice after treatment with 30 kBq [^225^Ac]Ac-SibuDAB or [^225^Ac]Ac-PSMA-617; Figure S6: Relative body mass of PC-3 PIP tumor-bearing mice treated with [^225^Ac]Ac-SibuDAB or [^225^Ac]Ac-PSMA-617; Figure S7: Comparison of the therapeutic efficacy of the radioligands in combination with actinium-225 and lutetium-177. Table S1: PSMA-binding affinity of the radioligands; Tables S2–S4: Single animal data of bone marrow and blood smears on Day 10, Day 28 and Day 56, respectively; Table S5: Scoring system for histopathological analysis; Table S6: Incidence and score average per microscopic finding in the spleen, salivary glands and kidneys; Tables S7–S10: Biodistribution data and tumor-to-background ratios obtained in PC3-PIP/flu tumor-bearing mice after injection of [^225^Ac]Ac-SibuDAB, [^177^Lu]Lu-SibuDAB, [^225^Ac]Ac-PSMA-617 or [^177^Lu]Lu-PSMA-617 at 1 h, 4 h, 24 and 48 h p.i., respectively; Table S11: Relative body mass and organ masses of mice that survived until study end; Table S12: Organ-to-brain mass ratios of mice treated with [^225^Ac]Ac-SibuDAB; Table S13: Blood chemistry values of mice treated with [^225^Ac]Ac-SibuDAB.
