# Supplementary material for: [225Ac]Ac-SibuDAB for Targeted Alpha Therapy of Prostate Cancer: Preclinical Evaluation and Comparison with [225Ac]Ac-PSMA-617
Source: Cancers (Basel). 2022 Nov 17;14(22):5651. doi: 10.3390/cancers14225651 (PMC9688344; doi:10.3390/cancers14225651)
Supplement: Supplementary file 1 [file cancers-14-05651-s001.zip › cancers-2027428-supplementary.pdf]

cancers

MDPI

Supplementary Material

[^{225}Ac]Ac-SibuDAB for Targeted Alpha Therapy of Prostate Cancer: Preclinical Evaluation and Comparison with [^{225}Ac]Ac-PSMA-617

Sarah D. Busslinger, Viviane J. Tschan, Olivia K. Richard, Zeynep Talip, Roger Schibli and Cristina Müller

1. Radiosynthesis of [^{225}Ac]Ac-SibuDAB and [^{225}Ac]Ac-PSMA-617

Purpose: SibuDAB and PSMA-617 were labeled with actinium-225 and the quality of the radioligands was assessed in order to determine the time period over which they remained intact and could be used for in vitro and in vivo experiments.

Methods: Radiolabeling of the PSMA ligands was performed at ITM Medical Isotopes GmbH, Munich, Germany. In brief, SibuDAB (29.2 µg corresponding to 20 nmol; 1 µg/µL in MilliQ water) and PSMA-617 (21 µg corresponding to 20 nmol; 1 µg/µL in MilliQ water) were labeled with 1 MBq [^{225}Ac]$AcCl_3$ (25 µL; 0.04 M HCl) in labeling buffer (100 µL; 3 M sodium acetate solution with 10% (*v*/*v*) ethanol) at pH ~7.4 to obtain a molar activity of 50 kBq/nmol. The reaction mixture was incubated for 8 min at 90 °C. The formulation buffer (40 mg/mL sodium ascorbate with 0.05% bovine serum albumin (BSA)), was added to obtain a final volume of 2.0 mL. Quality control was performed by thin-layer chromatography (TLC) using citrate buffer (0.1 M, pH 7.5) as mobile phase and a silica gel 60 f254 plate as stationary phase to separate free actinium-225 from DOTA-coordinated actinium-225 as the fraction which represented the radioligand. The developed TLC was let to decay for at least 7 h before reading using a TLC scanner (Scan-RAM radio-TLC scanner equipped with a PMT/Plastic scintillation detector from LabLogic, United Kingdom).

[^{225}Ac]Ac-SibuDAB and [^{225}Ac]Ac-PSMA-617 were shipped to the Paul Scherrer Institute (PSI) overnight. To assess the radioligands' quality over the 48 following hours upon arrival, TLC quality control was performed at PSI under the same experimental condition using a TLC scanner (miniGITA from Raytest, Germany).

Results: The fraction of intact radioligand determined by ITM Medical Isotopes GmbH immediately after radiolabeling and at PSI up to 2 days after arrival was >99%, indicating that the ^{225}Ac-labeled PSMA ligands remained entirely stable over this time period.

2. Measurement of Actinium-225

Purpose: In order to exactly determine the quantity of activity of ^{225}Ac-samples used for in vitro and in vivo studies, all dilutions of the respective radioligands were checked by activity measurement using γ-ray spectrometry at PSI. A calibration curve was established based on the counts measured in a γ-counter of samples with a defined activity determined by γ-ray spectrometry.

Methods: Calibration equations were determined based on the counts obtained from various activities measured in the γ-counter (using variable volumes of 100 µL, 200 µL, 300 µL, 500 µL 1000 µL or 2000 µL for the same activity) and the true activity values determined with the germanium detector (measured in a final volume of 1000 µL). These equations allowed to convert counts per minute (cpm) obtained in the γ-counter in Bq-values and enabled a faster determination of the activity in the samples under consideration of the volume effect. The samples of all experiments were measured when actinium-225 (mother nuclide) and the γ-emitting daughters had reached secular equilibrium. This

was the case after a minimum of 7 h when considering only the γ-emitting daughters. Samples obtained from the performed experiments (e.g. internalization studies, biodistribution studies) were commonly measured the following day, which means 16–24 h after completion of the experiment.

Results: The results of the actinium-225 measurements using a germanium detector had a standard deviation (SD) of 6–8%. The resultant calibration equations showed that the determination of the activity of ^{225}Ac-samples from the counts obtained in the γ-counter was dependent on the volume of the samples. The γ-counter determined fewer counts for a ^{225}Ac-sample of a larger volume than a sample with a smaller volume with the same activity. The activity was, therefore, determined in samples of 100 μL for which the activity (Bq) was calculated by multiplying the obtained cpm with the factor 0.033.

3. PBS/*n*-Octanol Distribution Coefficient (logD Values)

Purpose: The distribution coefficients (logD values) of [^{225}Ac]Ac-SibuDAB and [^{225}Ac]Ac-PSMA-617 were determined to obtain an indication of their lipophilic/hydrophilic properties and the results were compared to those of the respective ^{177}Lu-labeled counterparts.

Methods: The logD values of [^{225}Ac]Ac-SibuDAB and [^{225}Ac]Ac-PSMA-617 (~50 kBq/nmol) were determined as previously reported [1]. A sample of each ^{225}Ac-labeled radioligand (~50 kBq, 100 μL, ~1 nmol) was added to a mixture of 1400 μL phosphate-buffered saline (PBS) pH 7.4 and 1500 μL *n*-octanol. The vials were vortexed vigorously for 1 min followed by centrifugation for 6 min at 2500 rpm for phase separation. An aliquot was taken from each phase and the activity was measured in a γ-counter (Perkin Elmer, Wallac Wizard 1480). The distribution coefficients were calculated as the logarithm of the ratio of cpm measured in the *n*-octanol phase relative to the cpm measured in the PBS phase. The results were listed as mean ± SD of the data obtained from three independent experiments, each performed with five replicates. The results were compared to previously reported logD values of the ^{177}Lu-labeled counterparts [2,3].

Results: The results are presented in the main article.

4. Cell Uptake and Internalization

Purpose: The uptake and internalization of PSMA ligands labeled with actinium-225 were assessed and the data compared to previously published results of their respective ^{177}Lu-labled counterparts.

Methods: The PC-3 PIP and PC-3 flu tumor cells were seeded in 12-well plates (0.3 × 10^6 cells in 2 mL per well) using RPMI cell culture medium with supplements. The tumor cells were incubated overnight to allow adhesion. The cells were rinsed with PBS followed by the addition of the respective radioligand (~50 kBq/nmol; 375 Bq; 7.5 pmol) diluted in RPMI cell culture medium supplemented with 1% fetal calf serum (FCS). After incubation of the cells for 4 h at 37 °C, 5% CO_2, they were rinsed three times with ice-cold PBS to determine the total uptake of the radioligand. In order to assess the internalized fraction, ice-cold glycine buffer (pH 2.8) was applied to release PSMA-bound radioligands from the cell surface. Cell samples were lysed by addition of 1 mL NaOH solution (1 M) to each well. After counting the samples for activity in a γ-counter (Perkin Elmer, Wallac Wizard 1480), the protein concentration of each sample was determined for standardization (Micro BCA Protein Assay kit; Pierce, Thermo Scientific). The uptake and internalized fraction were expressed as the percentage of total added activity and presented as the average ± SD of n=3–4 independent experiments each performed in six replicates.

Results: The PC-3 PIP cell uptake of [^{225}Ac]Ac-SibuDAB and [^{177}Lu]Lu-SibuDAB was similar after a 4 h-incubation time (59 ± 1% and 53 ± 1%, respectively, p>0.05). The internalized fraction of [^{225}Ac]Ac-SibuDAB was somewhat higher than that of [^{177}Lu]Lu-SibuDAB (25 ± 5% and 15 ± 2%; p<0.05; Figure S1A). The uptake of [^{225}Ac]Ac-PSMA-617

and [177Lu]Lu-PSMA-617 was comparable (63 ± 5% and 60 ± 5%; p>0.05) and the internalized fraction was slightly higher for the 225Ac-labeled counterpart (19 ± 3% *vs.* 13 ± 2%; p>0.05; Figure S1B). Uptake in PSMA-negative PC-3 flu cells was <1% for all radioligands (data not shown).

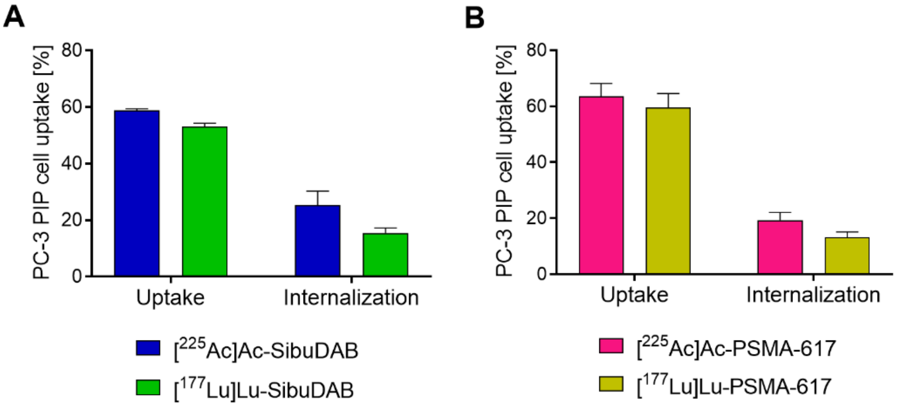

Figure S1. Cell uptake and internalization of (**A**) [225Ac]Ac-SibuDAB *vs.* [177Lu]Lu-SibuDAB and (**B**) [225Ac]Ac-PSMA-617 *vs.*[177Lu]Lu-PSMA-617 into PSMA-positive PC-3 PIP cells (average ± SD, n=3–4). Data points of [177Lu]Lu-SibuDAB and [177Lu]Lu-PSMA-617 were previously published by Borgna & Deberle et al., Mol Pharm 2022 [3] (Copyright 2022, American Chemical Society) and Tschan et al., Eur J Nucl Med Mol Imaging 2022 [4].

5. PSMA-Binding Affinity (K_D Values)

Purpose: The PSMA-binding affinity of the 225Ac-labeled PSMA ligands was assessed and compared to previously published results obtained for their respective 177Lu-labeled counterparts.

Methods: PSMA-positive PC-3 PIP cells were seeded in 48-well plates (8×10^4 cells/well) in 500 µL RPMI cell culture medium with supplements. The cells were incubated at 37 °C, 5% CO_2, to allow cell adhesion overnight. The experiment was performed on ice using ice-cold cell culture medium and buffer solutions. After removal of the supernatant, the cells were rinsed once with PBS prior to the addition of RPMI cell culture medium without supplements (450 µL/well). The respective radioligand (~50 kBq/nmol) was added to each well at variable concentrations (10–1000 nM, 50 µL per well). In order to determine the unspecific binding, some of the cell samples were co-incubated with excess 2-(phosphonomethyl)-pentanedioic acid (2-PMPA; 400 µM) to block PSMA on the cell surface. The PC-3 PIP tumor cells were incubated on ice while shaking for 30 min, followed by removal of the supernatants and rinsing the cells twice with PBS. The cells were lysed with 600 µL aqueous NaOH solution (1 M). The activity of each sample was counted in a γ-counter (Wallac Wizard 1480, Perkin Elmer) whereof cpm of the specific binding (determined by subtracting the cpm of the unspecific binding from the cpm of total binding), were plotted against the molar concentration of the added radioligand. The nonlinear regression analysis for determination of the K_D value was performed using GraphPad Prism software (version 8).

Results: The PSMA-binding affinities of radiolabeled PSMA-617 were similar, irrespective of whether it was labeled with actinium-225 or lutetium-177. The values for the 225Ac- and 177Lu-labeled SibuDAB, respectively, differed more but were still in the same range (Table S1).

Table S1. PSMA-binding affinity expressed as K_D values determined for SibuDAB and PSMA-617 labeled with actinium-225 or lutetium-177.

Radioligand	K_D Value [nM]	95% Confidence interval	n
[^{225}Ac]Ac-SibuDAB	17	11–23	4
[^{177}Lu]Lu-SibuDAB[1]	27	18–35	4
[^{225}Ac]Ac-PSMA-617	11	6–16	5
[^{177}Lu]Lu-PSMA-617[2]	13	9–17	3

[1] Data of [^{177}Lu]Lu-SibuDAB were previously published by Borgna & Deberle et al., Mol Pharm 2022 [3]; Copyright 2022, American Chemical Society.

[2] Data of [^{177}Lu]Lu-PSMA-617 were previously published by Deberle & Benešová et al., Theranostics 2020 [1].

6. Albumin-Binding of [^{225}Ac]Ac-SibuDAB and [^{177}Lu]Lu-SibuDAB

Purpose: The affinity of [^{225}Ac]Ac-SibuDAB to serum albumin in mouse and human blood plasma was assessed and compared to that of [^{177}Lu]Lu-SibuDAB to determine the impact of the radionuclide on the albumin-binding properties of SibuDAB.

Methods: Relative albumin-binding affinities were determined using an ultrafiltration assay according to a slightly adapted version of a published protocol [5]. The concentration of mouse serum albumin (MSA) and human serum albumin (HSA) in samples of mouse and human plasma, respectively, was determined using a DRI-CHEM Analyzer (FUJIFILM, 4000i). These mouse and human blood plasma samples were diluted with PBS to obtain MSA concentrations in the range of 0.4–500 µM and HSA concentrations in the range of 0.4–800 µM, respectively. A fixed amount of the radioligand (15 µL, 60 pmol) was added to these dilutions (150 µL). A molar activity of 50 kBq/nmol for [^{225}Ac]Ac-SibuDAB and 5 MBq/nmol for [^{177}Lu]Lu-SibuDAB was chosen to obtain results within the linear detection range of the γ-counter (Perkin Elmer Wallac Wizard 1480), allowing to keep the amount of total ligand added to the plasma dilutions constant. The mixtures were incubated for 30 min at 37 °C. Afterwards, the samples were cooled on ice followed by addition of 150 µL ice-cold PBS. The samples were transferred to Amicon centrifugal filters (molecular weight cut-off = 10 kDa) and centrifuged (14000 rcf) for 30 min at 4 °C. The filter inserts were inverted and centrifuged (200 rcf) for 3 min to recover the fraction bound to the proteins. The activity in the retained protein fraction (A_{bound}), in the filtrate ($A_{filtrate}$) and in the filter unit (A_{filter}) were measured separately in a γ-counter (Perkin Elmer Wallac Wizard 1480). The total activity (A_{total}) was determined as the sum of counted activity in all three fractions ($A_{bound} + A_{filtrate} + A_{filter}$). The percentage of radioligand bound to mouse or human plasma proteins was calculated (bound radioligand = $A_{bound}/A_{total} \times 100$). The results were presented as average ± standard deviation (SD) of n=3–4 independent experiments. The percentage of radioligand bound to mouse or human plasma (A_{bound}) was plotted against the molar ratios of MSA/HSA to radioligand and fitted with a nonlinear regression curve (specific binding with Hill slope, B_{max} set to 100%) using GraphPad Prism software (version 8) to obtain the half maximum binding (B_{50}).

Results: The results are reported in the main article.

7. Tolerability Study

Purpose: A tolerability study with normal, immunocompetent FVB mice was conducted in order to assess potential side effects to the bone marrow, spleen, salivary glands and kidneys after the application of high activities of the ^{225}Ac-labeled PSMA radioligands.

Methods: *Monitoring Body Mass and Signs of Unease:* Mice were monitored by measuring their body mass (BM) three times a week. The body masses were expressed as the relative body mass, defined as [BM_x/BM_0], where BM_x is the body mass in grams on a given Day x and BM_0 is the body mass in grams on Day 0. Endpoint criteria, defined as (i) >15% loss in body mass, (ii) signs of unease and pain or a combination thereof based on a scoring system, were not reached in this study.

Hematology: Blood cell counts were determined in blood samples taken by cardiac puncture immediately after euthanasia and collected in EDTA tubes (Microvette® 100 K3 EDTA, Sarstedt, Germany). Leukocyte, lymphocyte, erythrocyte and thrombocyte counts as well as hemoglobin and hematocrit values were determined using a hematology analyzer (VetScan HM5, Abaxis, United States). Blood smears were prepared on glass slides with cardiac blood. The bone marrow was collected from the femur, diluted in FCS/H_2O (2:1 *v*/*v*) and smeared onto microscopy slides using a paintbrush. The slides were stained with a May Grünwald solution and subsequently with diluted Giemsa solution using the Pappenheim method [6] and covered with coverslips using mounting medium (Pertex). The assessment of potential morphological changes of the blood cells and counting of the subgroups of leukocytes was performed by a board-certified veterinary pathologist (AnaPath Services GmbH, Liestal, Switzerland).

Histopathological Analysis: Spleen, salivary glands and kidneys were formalin-fixed and paraffin-embedded. Tissue sections were stained with hematoxylin & eosin (H&E) and analyzed.

Blood Plasma Parameters: Blood plasma parameters were determined in plasma obtained from centrifuged retrobulbar blood samples collected in lithium-heparin tubes (Microvette® 200 LH, SARSTEDT). The concentrations of blood urea nitrogen (BUN), alkaline phosphatase (ALP), total bilirubin (TBIL) and albumin (ALB) were determined using a dry chemistry analyzer (DRI-CHEM 4000i, FUJIFILM, Japan).

Results: The results of the tolerability study are described in the main article and presented in Figures S2-S5 and Tables S2-S6.

Body Mass: The average relative body mass of mice from each group increased until study end on Day 56. No major differences in the relative body masses were observed between mice treated with ^{225}Ac-based radioligands and untreated controls (Figure S2).

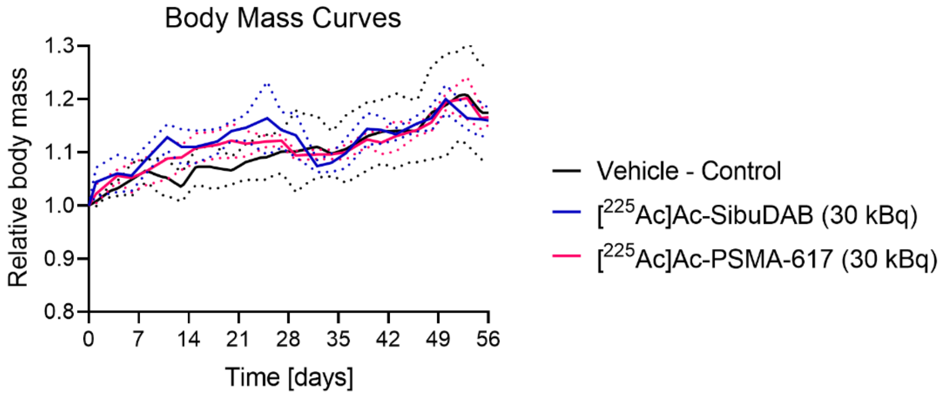

Figure S2. Relative body mass of non-tumor-bearing FVB mice that received only vehicle (controls) or 30 kBq [^{225}Ac]Ac-SibuDAB or 30 kBq [^{225}Ac]Ac-PSMA-617.

Blood Cell Counts: The results of the leukocyte, lymphocyte, erythrocyte and thrombocyte counts are described and discussed in the main article (Figure 3). Decreases in hematocrit and hemoglobin values of mice treated with [^{225}Ac]Ac-SibuDAB or [^{225}Ac]Ac-PSMA-617 as compared to control mice were associated with a decline in erythrocytes on Day 28 (Figure S3). On Day 56, erythrocyte counts as well as hematocrit and hemoglobin values of mice that received either of the ^{225}Ac-based radioligands were elevated as compared to untreated controls.

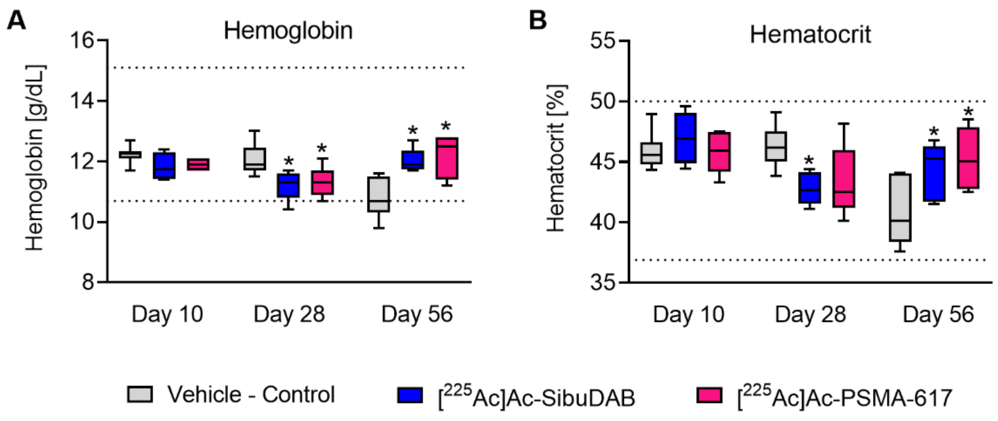

Figure S3. Hematological analysis of the blood from mice (*n*=4–7) on Day 10, 28 and 56 after treatment with vehicle (control) or 30 kBq [^{225}Ac]Ac-SibuDAB or 30 kBq [^{225}Ac]Ac-PSMA-617. (**A**) Hemoglobin and (**B**) Hematocrit. Values that are significantly different from those of control mice are indicated with an asterisk (*). The dotted lines represent reference values for female FVB mice previously reported by Schneck et al. [7].

Blood and Bone Marrow Smears: On Day 10, one out of five mice treated with [^{225}Ac]Ac-SibuDAB and two out of five mice injected with [^{225}Ac]Ac-PSMA-617 showed a relative increase of neutrophilic blasts (metamyelocytes) in the peripheral blood. At this timepoint, the analysis of the bone marrow smears revealed a relative decrease in erythropoiesis with a relative increase in myelopoiesis for mice that were injected with [^{225}Ac]Ac-SibuDAB or [^{225}Ac]Ac-PSMA-617 (66% and 62% myelopoiesis, respectively) as compared to control mice (58% myelopoiesis; Table S2). The increase in myelopoiesis was associated with an increased production of granulocytes. On Day 28, the ratio changed towards the erythropoietic precursor cells and normalized on Day 56 (Table S3, S4).

Table S2. Single animal data of bone marrow and blood smears on Day 10.

		Bone marrow			Blood
		% E	% M	Notes	Notes
Vehicle – Control	M1	40	60	-	-
	M2	40	60	-	-
	M3	40	60	-	-
	M4	50	50	-	More neutrophilic granulocytes, mostly segmented
	M5	40	60	-	-
[^{225}Ac]Ac-SibuDAB	M1	30	70	More blasts, neutrophilic row	-
	M2	50	50	-	-
	M3	40	60	-	-
	M4	30	70	More blasts, neutrophilic row	More neutrophilic blasts (Metamyelocyte)
	M5	20	80	More blasts, neutrophilic row	-
[^{225}Ac]Ac-PSMA-617	M1	50	50	-	More neutrophilic blasts (Metamyelocyte)
	M2	60	40	-	More neutrophilic blasts (Metamyelocyte)
	M3	20	80	More blasts, neutrophilic row	-
	M4	30	70	More blasts, neutrophilic row	-
	M5	30	70	More blasts, neutrophilic row	-

Table S3. Single animal data of bone marrow and blood smears on Day 28.

		Bone marrow			Blood
		% E	% M	Notes	Notes
Vehicle – Control	M1	40	60	-	-
	M2	40	60	-	-
	M3	40	60	-	-
	M4	40	60	-	-
	M5	50	50	-	More neutrophilic granulocytes, mostly segmented
[225Ac]Ac-SibuDAB	M1	40	60	-	-
	M2	50	50	-	-
	M3	40	60	-	-
	M4	50	50	-	-
	M5	50	50	-	-
[225Ac]Ac-PSMA-617	M1	50	50	-	-
	M2	40	60	-	-
	M3	40	60	-	-
	M4	50	50	-	-
	M5	40	60		

Table S4. Single animal data of bone marrow and blood smears on Day 56.

		Bone marrow			Blood
		% E	% M	Notes	Notes
Vehicle – Control	M1	40	60	-	-
	M2	40	60	-	-
	M3	40	60	-	-
	M4	40	60	-	-
	M5	40	60	-	-
[225Ac]Ac-SibuDAB	M1	40	60	-	-
	M2	30	70	-	-
	M3	40	60	-	-
	M4	40	60	-	-
	M5	40	60	-	-
[225Ac]Ac-PSMA-617	M1	30	70	-	-
	M2	40	60	-	-
	M3	30	70	-	-
	M4	40	60	-	-

Histopathological Analysis of the Spleen, Salivary Glands and Kidneys: A pre-defined scoring system was applied to analyze the histological findings (Table S5).

Table S5. Scoring system for histopathological analysis of selected tissues.

Score	Definition
Score 1<br>Minimal	Minor, small or infrequent histopathologic changes ranging from inconspicuous to barely noticeable. For multifocal or diffusely-distributed lesions, this grade was used for processes in which less than approximately 10% of the tissue in an average high-power field was involved.
Score 2<br>Slight	Histopathologic changes that are noticeable but not a prominent feature of the tissue. For multifocal or diffusely-distributed lesions, this grade was used for processes in which approximately 10% to 25% of the tissue in an average high-power field was involved.
Score 3<br>Moderate	Histopathologic changes that are prominent but not dominant features of the tissue. For multifocal or diffusely-distributed lesions, this grade was used for processes in which approximately 25% to 50% of the tissue in an average high-power field was involved.
Score 4<br>Marked	Histopathologic changes that are dominant but not an overwhelming feature of the tissue. For multifocal or diffusely-distributed lesions, this grade was used for processes in which approximately 50% to 95% of the tissue in an average high-power field was involved.
Score 5<br>Severe (Massive)	Histopathologic changes that are an overwhelming feature of the tissue. For multifocal or diffusely-distributed lesions, this grade was used for processes in which more than approximately 95% of the tissue in an average high-power field was involved.

The spleen, as an organ of major hematopoiesis, revealed a minimal to mild lymphoid hyperplasia on Day 28 in all mice that received either of the ^{225}Ac-based radioligands which was, however, not visible in mice that were euthanized on Day 10 or Day 56 (Figure S4, Table S6).

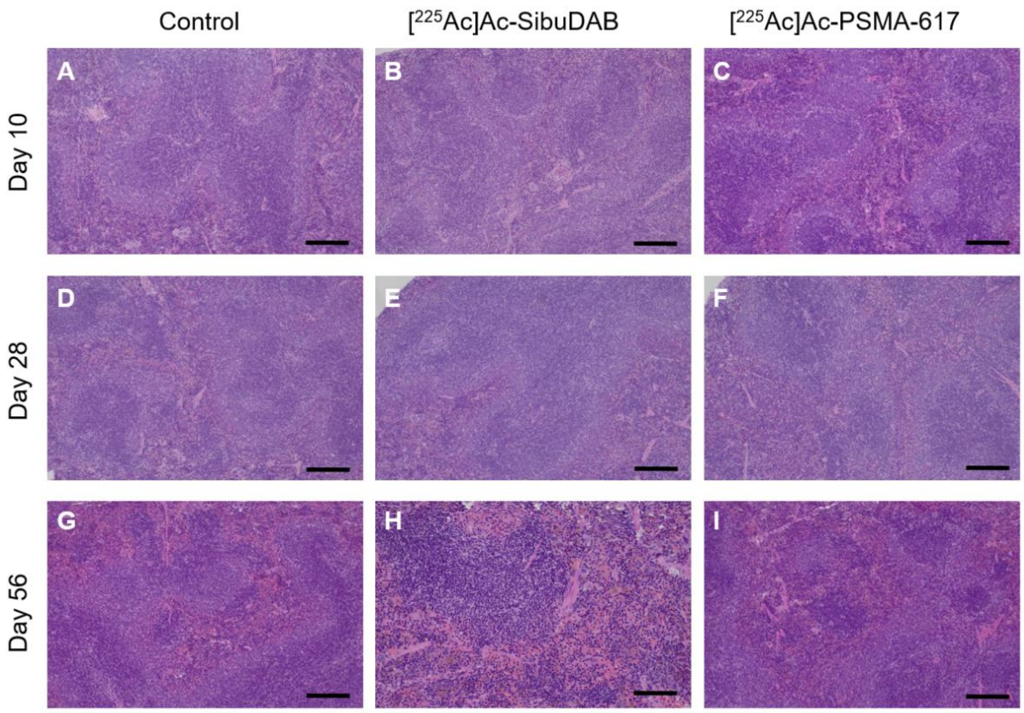

Figure S4. Representative images of H&E stained paraffin-embedded sections of the spleen on Day 10, Day 28 or Day 56 after vehicle or radioligand injection. (**A**,**D**,**G**) vehicle (control); (**B**,**E**,**H**) [^{225}Ac]Ac-SibuDAB; (**C**,**F**,**I**) [^{225}Ac]Ac-PSMA-617.

Histological investigations of the salivary glands are reported in the main article. The analysis of kidney tissue revealed only background lesions with no major differences between control kidneys and those of mice that received one of the radioligands (Table S6).

Table S6. Incidence and score average per microscopic finding in the spleen, salivary glands and kidneys on Day 10, Day 28 and Day 56.

	**Control**			**[^{225}Ac]Ac-SibuDAB**			**[^{225}Ac]Ac-PSMA-617**		
				**Spleen**					
**Day**	**10**	**28**	**56**	**10**	28	56	10	28	56
	I/A	I/A	I/A	I/A	I/A	I/A	I/A	I/A	I/A
Extramedullary hematopoiesis	5/1.2	5/1.8	4/1.5	5/1.2	5/1.8	5/1.8	5/1.0	5/1.8	5/1.4
Pigment	5/1.0	5/2.0	4/2.0	5/1.0	5/2.0	5/2.0	5/1.0	5/1.8	5/2.0
Hyperplasia, lymphoid	0/0.0	0/0.0	0/0.0	1/1.0	5/1.2	0/0.0	1/1.0	5/1.0	0/0.0
				**Salivary glands**					
Day	10	28	56	10	28	56	10	28	56
	I/A	I/A	I/A	I/A	I/A	I/A	I/A	I/A	I/A
Mononuclear cell foci	1/1.0	2/1.0	0/0.0	1/1.0	3/1.0	2/1.0	1/1.0	1/1.0	2/1.0
Hypertrophy acinar, sublingual	0/0.0	0/0.0	0/0.0	0/0.0	1/1.0	0/0.0	0/0.0	0/0.0	0/0.0
Ductal hyperplasia with inflammatory cells	0/0.0	0/0.0	0/0.0	0/0.0	0/0.0	0/0.0	0/0.0	0/0.0	0/0.0
Necrosis, multifocal, serous acinar cells	0/0.0	0/0.0	0/0.0	2/1.0	0/0.0	0/0.0	2/1.0	5/1.8	0/0.0
Vacuolation	0/0.0	0/0.0	1/1.0	1/1.0	0/0.0	0/0.0	0/0.0	0/0.0	0/0.0
				**Kidneys**					
Day	10	28	56	10	28	56	10	28	56
	I/A	I/A	I/A	I/A	I/A	I/A	I/A	I/A	I/A
Inflammatory cells, pelvis	0/0.0	1/1.0	0/0.0	0/0.0	2/1.0	1/1.0	0/0.0	0/0.0	0/0.0
Inflammatory cells, interstitium	0/0.0	1/1.0	0/0.0	0/0.0	1/1.0	1/1.0	0/0.0	0/0.0	0/0.0
Tubular basophilia	0/0.0	0/0.0	0/0.0	0/0.0	0/0.0	2/1.0	0/0.0	1/1.0	1/1.0
Hyperplasia/ hypertrophy, tubular	0/0.0	0/0.0	2/1.0	0/0.0	0/0.0	1/1.0	2/1.0	1/1.0	1/1.0
Mineralization, papilla	0/0.0	0/0.0	0/0.0	0/0.0	0/0.0	0/0.0	0/0.0	1/1.0	0/0.0
Hypertrophy/hyperplasia, tubular with ectasia	0/0.0	0/0.0	1/1.0	0/0.0	0/0.0	0/0.0	0/0.0	0/0.0	0/0.0

I = incidence, A = average, 1-5= score.

Blood Plasma Chemistry: On Day 10 after treatment, no statistically significant differences between the blood plasma parameters of the control group and those of mice treated with the PSMA radioligands were determined (p>0.05; Figure S5). On Day 28, blood urea nitrogen levels of mice injected with [^{225}Ac]Ac-SibuDAB (9.1 ± 1.1 mmol/L) and [^{225}Ac]Ac-PSMA-617 (8.7 ± 1.1 mmol/L) were similar (p>0.05), although in the case of [^{225}Ac]Ac-SibuDAB significantly elevated as compared to those of control mice of the same age (6.7 ± 0.7 mmol/L, p<0.05). This was, however, no longer the case on Day 56 (Figure S5A). Irrespective of the treatment, alkaline phosphatase levels decreased while total bilirubin increased with the age of the mice, but remained comparable among treated mice and untreated controls at all three investigated timepoints (p>0.05; Figures S5B,C). Albumin concentrations were equal in all groups of mice on Day 10 and Day 56, but temporarily elevated in mice treated with [^{225}Ac]Ac-SibuDAB (26 ± 1 g/L; p<0.05) on Day 28 as compared to those of control mice (24 ± 3 g/L) and mice that received [^{225}Ac]Ac-PSMA-617 (23 ± 2 g/L) (Figure S5D).

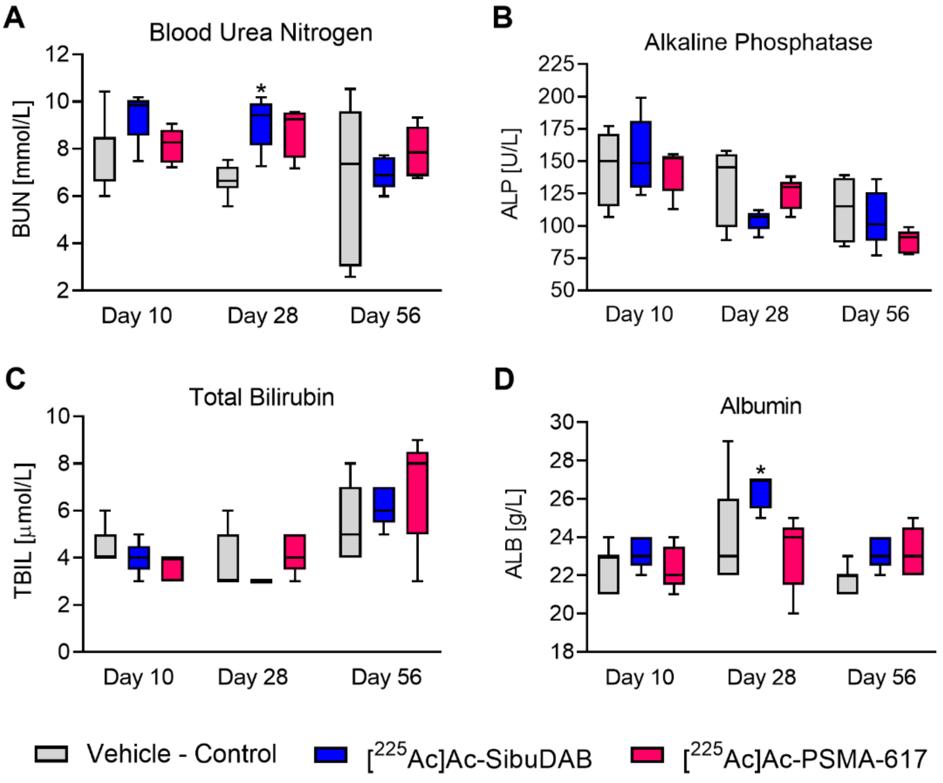

Figure S5. Blood plasma chemistry of mice on Day 10, 28 and 56 after treatment with vehicle (controls) or 30 kBq [^{225}Ac]Ac-SibuDAB or [^{225}Ac]Ac-PSMA-617. (**A**) Blood urea nitrogen (BUN); (**B**) Alkaline phosphatase (ALP); (**C**) Total bilirubin (TBIL); (**D**) Albumin (ALB). Values that were significantly different from those of control mice were highlighted with an asterisk (*).

8. Biodistribution Studies

Purpose: The tissue distribution profiles of [^{225}Ac]Ac-SibuDAB and [^{225}Ac]Ac-PSMA-617 were compared in PC-3 PIP/flu tumor-bearing mice. Furthermore, the data were compared to previously published results obtained for the PSMA ligands radiolabeled with lutetium-177 [2,3].

Methods: Five- to six-week-old female, athymic BALB/c nude mice were purchased from Charles River Laboratories (Sulzfeld, Germany). After an acclimatization period of at least 7 days, the mice were subcutaneously inoculated with PSMA-positive PC-3 PIP tumor cells (6×10^6 cells in 100 µL Hanks' balanced salt solution (HBSS)) on the right shoulder and PSMA-negative PC-3 flu tumor cells (5×10^6 cells in 100 µL HBSS) on the left shoulder as previously reported [8]. Approximately two weeks later, biodistribution studies were performed after intravenous administration of the radioligands. The ^{225}Ac-labeled ligands (40 kBq; 1 nmol, 100 µL saline containing 0.05% BSA per mouse) were injected into the tail vein using n=4–5 mice per timepoint. At 1 h, 4 h, 24 h and 48 h post injection (p.i.), the mice were sacrificed and selected tissues and organs were collected, weighed and counted 24 h later for activity using a γ-counter (PerkinElmer Wallac Wizard 1480). The results were reported as the percentage of the injected activity per gram of tissue mass (% IA/g) using counts of standards (5%, 10% and 20% of the injected activity for ^{225}Ac-labeled radioligands) measured at the same time to obtain decay-corrected data. The results were compared to data previously obtained under the same experimental conditions for [^{177}Lu]Lu-SibuDAB [3] and [^{177}Lu]Lu-PSMA-617 [2].

Results: Biodistribution data obtained at 1 h, 4 h, 24 h and 48 h after injection of the radioligands showed enhanced blood retention and significantly increased tumor uptake for [^{225}Ac]Ac-SibuDAB in comparison to [^{225}Ac]Ac-PSMA-617 (Tables S7–S10). Importantly, the retention of [^{225}Ac]Ac-SibuDAB in blood, kidneys and PC-3 PIP tumor were

different from the uptake of [^{177}Lu]Lu-SibuDAB in these same tissues. Additional results are reported in the main article.

Table S7. Biodistribution data and tumor-to-background ratios obtained in PC3-PIP/flu tumor-bearing mice 1 h after injection of [^{225}Ac]Ac-SibuDAB, [^{177}Lu]Lu-SibuDAB, [^{225}Ac]Ac-PSMA-617 or [^{177}Lu]Lu-PSMA-617. Decay-corrected data of accumulated activity are shown as [% IA/g]-values, representing the average ± SD.

	**Biodistribution data at 1 h p.i.**			
	[^{225}Ac]Ac-SibuDAB	[^{177}Lu]Lu-SibuDAB[1]	[^{225}Ac]Ac-PSMA-617	[^{177}Lu]Lu-PSMA-617[2]
	n = 5	*n* = 9	*n* = 4	*n* = 3
Blood	23 ± 2	17 ± 2	0.62 ± 0.12	0.50 ± 0.06
Heart	7.4 ± 0.9	5.3 ± 0.8	0.35 ± 0.09	0.29 ± 0.03
Lung	19 ± 2	12 ± 2	0.76 ± 0.23	0.57 ± 0.07
Spleen	3.5 ± 0.3	3.0 ± 0.4	0.68 ± 0.09	0.63 ± 0.27
Kidneys	18 ± 1	22 ± 5	12 ± 3	9.8 ± 1.4
Stomach	2.4 ± 0.4	1.5 ± 0.4	0.37 ± 0.14	0.18 ± 0.02
Intestines	2.6 ± 0.5	1.8 ± 0.3	0.29 ± 0.12	0.24 ± 0.01
Liver	4.4 ± 0.4	3.5 ± 0.6	0.46 ± 0.10	0.20 ± 0.04
Salivary glands	4.7 ± 0.4	3.5 ± 0.4	0.30 ± 0.06	0.42 ± 0.30
Muscle	2.0 ± 0.4	1.9 ± 0.3	0.18 ± 0.06	0.25 ± 0.20
Bone	2.5 ± 0.2	2.1 ± 0.3	0.46 ± 0.13	0.21 ± 0.04
PC-3 PIP tumor	20 ± 2	38 ± 7	46 ± 13	44 ± 12
PC-3 flu tumor	4.2 ± 1.1	2.7 ± 0.4	0.30 ± 0.06	0.28 ± 0.04
Tumor-to-blood	0.9 ± 0.1	2.3 ± 0.5	73 ± 7	88 ± 13
Tumor-to-liver	4.6 ± 0.5	11 ± 2	99 ± 8	213 ± 17
Tumor-to-kidney	1.2 ± 0.1	1.7 ± 0.3	3.7 ± 0.3	4.5 ± 0.7

[1] Data of [^{177}Lu]Lu-SibuDAB were previously published by Borgna & Deberle et al., Mol Pharm 2022 [3]; Copyright 2022, American Chemical Society.
[2] Data of [^{177}Lu]Lu-PSMA-617 were previously published by Benešová et al., Mol Pharm 2018 [2]; Copyright 2018, American Chemical Society.

Table S8. Biodistribution data and tumor-to-background ratios obtained in PC3-PIP/flu tumor-bearing mice 4 h after injection of [^{225}Ac]Ac-SibuDAB, [^{177}Lu]Lu-SibuDAB, [^{225}Ac]Ac-PSMA-617 or [^{177}Lu]Lu-PSMA-617. Decay-corrected data of accumulated activity are shown as [% IA/g]-values, representing the average ± SD.

	**Biodistribution data at 4 h p.i.**			
	[^{225}Ac]Ac-SibuDAB	[^{177}Lu]Lu-SibuDAB[1]	[^{225}Ac]Ac-PSMA-617	[^{177}Lu]Lu-PSMA-617[2]
	n = 4	*n* = 6	*n* = 4	*n* = 3
Blood	16 ± 2	2.9 ± 1.9	≤ 0.10	≤ 0.10
Heart	5.5 ± 0.7	1.1 ± 0.7	≤ 0.10	≤ 0.10
Lung	10 ± 2	3.1 ± 2.8	≤ 0.10	≤ 0.10
Spleen	3.0 ± 0.5	1.1 ± 0.6	0.12 ± 0.05	0.15 ± 0.04
Kidneys	19 ± 1	12 ± 3	3.5 ± 0.6	3.7 ± 1.1
Stomach	1.8 ± 0.3	0.49 ± 0.29	≤ 0.10	≤ 0.10
Intestines	2.2 ± 0.3	0.46 ± 0.23	≤ 0.10	≤ 0.10
Liver	4.0 ± 0.3	1.3 ± 0.5	0.31 ± 0.04	≤ 0.10
Salivary glands	3.7 ± 0.2	0.82 ± 0.43	≤ 0.10	≤ 0.10
Muscle	1.9 ± 0.2	0.39 ± 0.22	≤ 0.10	≤ 0.10
Bone	2.2 ± 0.5	0.49 ± 0.27	0.14 ± 0.04	≤ 0.10
PC-3 PIP Tumor	45 ± 5	66 ± 11	43 ± 10	56 ± 8
PC-3 flu Tumor	4.1 ± 0.8	0.95 ± 0.71	0.11 ± 0.02	≤ 0.10
Tumor-to-blood	2.9 ± 0.3	49 ± 51	2593 ± 825	2315 ± 108
Tumor-to-liver	11 ± 1	61 ± 29	142 ± 23	598 ± 108
Tumor-to-kidney	2.3 ± 0.1	5.5 ± 1.5	13 ± 4	16 ± 2

[1] Data of [^{177}Lu]Lu-SibuDAB were previously published by Borgna & Deberle et al., Mol Pharm 2022 [3]; Copyright 2022, American Chemical Society.

[2] Data of [^{177}Lu]Lu-PSMA-617 were previously published by Benešová et al., Mol Pharm 2018 [2]; Copyright 2018, American Chemical Society.

Table S9. Biodistribution data and tumor-to-background ratios obtained in PC3-PIP/flu tumor-bearing mice 24 h after injection of [^{225}Ac]Ac-SibuDAB, [^{177}Lu]Lu-SibuDAB, [^{225}Ac]Ac-PSMA-617 or [^{177}Lu]Lu-PSMA-617. Decay-corrected data of accumulated activity are shown as [% IA/g]-values, representing the average ± SD.

	**Biodistribution data at 24 h p.i.**			
	[^{225}Ac]Ac-SibuDAB	[^{177}Lu]Lu-SibuDAB[1]	[^{225}Ac]Ac-PSMA-617	[^{177}Lu]Lu-PSMA-617[2]
	n = 5	*n* = 8	*n* = 4	*n* = 3
Blood	3.3 ± 0.7	0.26 ± 0.08	≤ 0.10	≤ 0.10
Heart	1.5 ± 0.4	0.15 ± 0.05	≤ 0.10	≤ 0.10
Lung	2.9 ± 0.4	0.31 ± 0.10	≤ 0.10	≤ 0.10
Spleen	2.3 ± 0.5	0.40 ± 0.13	0.13 ± 0.04	≤ 0.10
Kidneys	18 ± 2	4.3 ± 1.0	1.2 ± 0.2	0.76 ± 0.15
Stomach	0.72 ± 0.14	0.12 ± 0.06	≤ 0.10	≤ 0.10
Intestines	0.85 ± 0.14	0.13 ± 0.06	≤ 0.10	≤ 0.10
Liver	3.3 ± 0.4	0.54 ± 0.18	0.39 ± 0.06	≤ 0.10
Salivary glands	1.7 ± 0.2	0.18 ± 0.05	≤ 0.10	≤ 0.10
Muscle	0.58 ± 0.06	≤ 0.10	≤ 0.10	≤ 0.10
Bone	0.81 ± 0.10	0.11 ± 0.04	0.11 ± 0.01	≤ 0.10
PC-3 PIP tumor	80 ± 8	62 ± 18	37 ± 9	37 ± 6
PC-3 flu tumor	2.4 ± 0.9	0.38 ± 0.09	0.11 ± 0.08	≤ 0.10
Tumor-to-blood	25 ± 6	247 ± 34	4991 ± 3208	2730 ± 195
Tumor-to-liver	25 ± 5	117 ± 14	97 ± 20	528 ± 51
Tumor-to-kidney	4.7 ± 0.9	14 ± 1	33 ± 9	50 ± 4

[1] Data of [^{177}Lu]Lu-SibuDAB were previously published by Borgna & Deberle et al., Mol Pharm 2022 [3]; Copyright 2022, American Chemical Society.

[2] Data of [^{177}Lu]Lu-PSMA-617 were previously published by Benešová et al., Mol Pharm 2018 [2]; Copyright 2018, American Chemical Society.

Table S10. Biodistribution data and tumor-to-background ratios obtained in PC3-PIP/flu tumor-bearing mice 48 h after injection of [^{225}Ac]Ac-SibuDAB, [^{177}Lu]Lu-SibuDAB, [^{225}Ac]Ac-PSMA-617 or [^{177}Lu]Lu-PSMA-617. Decay-corrected data of accumulated activity are shown as [% IA/g]-values, representing the average ± SD.

	Biodistribution data at 48 h p.i.			
	[^{225}Ac]Ac-SibuDAB	[^{177}Lu]Lu-SibuDAB	[^{225}Ac]Ac-PSMA-617	[^{177}Lu]Lu-PSMA-617[1]
	n = 4	*n* = 4	*n* = 4	*n* = 3
Blood	1.1 ± 0.2	0.27 ± 0.04	≤ 0.10	≤ 0.10
Heart	0.73 ± 0.17	0.14 ± 0.02	≤ 0.10	≤ 0.10
Lung	1.3 ± 0.3	0.33 ± 0.07	≤ 0.10	≤ 0.10
Spleen	1.9 ± 0.5	0.43 ± 0.10	0.13 ± 0.02	≤ 0.10
Kidneys	8.6 ± 1.1	3.5 ± 0.7	0.79 ± 0.13	0.35 ± 0.05
Stomach	0.38 ± 0.04	0.12 ± 0.06	≤ 0.10	≤ 0.10
Intestines	0.40 ± 0.08	0.10 ± 0.03	≤ 0.10	≤ 0.10
Liver	2.7 ± 0.5	0.44 ± 0.06	0.44 ± 0.16	≤ 0.10
Salivary glands	0.92 ± 0.19	0.18 ± 0.05	≤ 0.10	≤ 0.10
Muscle	0.30 ± 0.11	0.07 ± 0.02	≤ 0.10	≤ 0.10
Bone	0.48 ± 0.09	≤ 0.10	0.12 ± 0.04	≤ 0.10
PC-3 PIP tumor	64 ± 11	69 ± 12	31 ± 3	28 ± 4
PC-3 flu tumor	1.5 ± 0.4	0.30 ± 0.07	0.26 ± 0.4	≤ 0.10
Tumor-to-blood	62 ± 11	252 ± 24	1716 ± 860	3776 ± 585
Tumor-to-liver	24 ± 4	157 ± 14	68 ± 15	710 ± 97
Tumor-to-kidney	7.4 ± 1.3	20 ± 2	37 ± 6	81 ± 11

[1] Data of [^{177}Lu]Lu-PSMA-617 were previously published by Benešová et al., Mol Pharm 2018 [2]; Copyright 2018, American Chemical Society.

9. Therapy Study

Purpose: In order to determine the therapeutic efficacy of [^{225}Ac]Ac-SibuDAB in comparison to [^{225}Ac]Ac-PSMA-617 as well as the ^{177}Lu-labeled counterparts, therapy studies were conducted in PC-3 PIP xenograft-bearing BALB/c nude mice.

Methods: The methods of the therapy study to assess the therapeutic efficacy of [^{225}Ac]Ac-SibuDAB are reported in the main manuscript. An additional group of mice was treated with 10 MBq (1 nmol) [^{177}Lu]Lu-SibuDAB under the same experimental conditions. Data previously acquired by our group for mice treated with 5 MBq [^{177}Lu]Lu-SibuDAB were published by Borgna & Deberle et al. [3] and data of mice treated with 5 MBq or 10 MBq [^{177}Lu]Lu-PSMA-617 were previously published by Tschan et al. [9] and Umbricht et al. [8], respectively.

Results: The assessment of the PSMA-specific therapeutic effect of [^{225}Ac]Ac-SibuDAB as well as the comparison to [^{225}Ac]Ac-PSMA-617 are described in the main article. The body mass of effectively treated mice, in which the tumor disappeared entirely or shrank, increased over the course of the study while body mass loss was observed for tumor-bearing mice that were treated with only vehicle as well as for some mice that were treated with 5 kBq or 10 kBq [^{225}Ac]Ac-PSMA-617 and experienced a relapse in tumor growth (Figure S6).

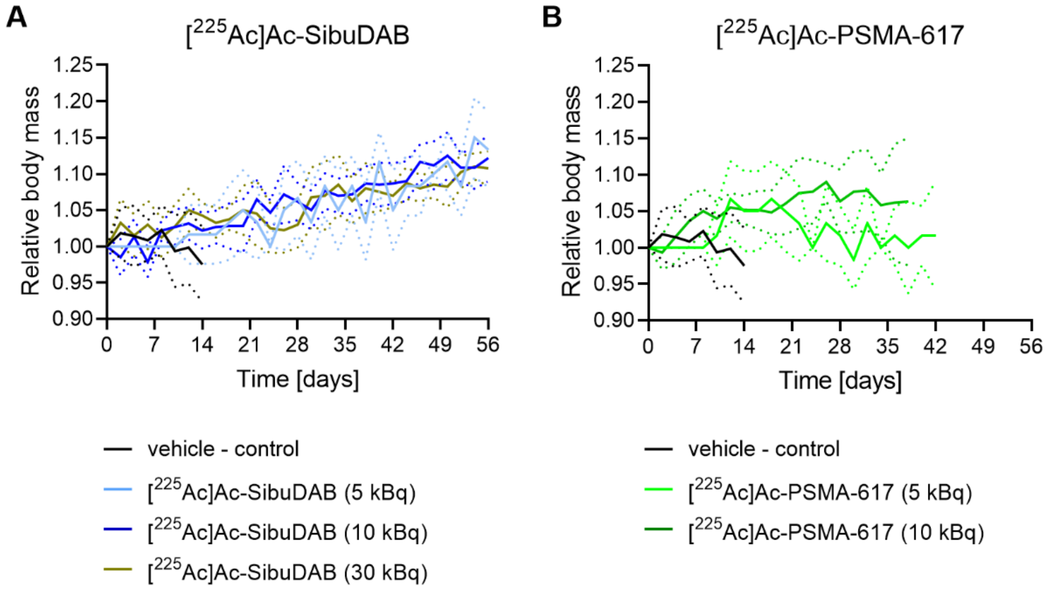

Figure S6. Relative body mass of mice bearing PC-3 PIP tumor xenografts treated with (**A**) [^{225}Ac]Ac-SibuDAB or (**B**) [^{225}Ac]Ac-PSMA-617 as compared to mice injected with vehicle as control. The curves represent the average ± SD and are shown until the first mouse of the respective group reached an endpoint. Data of control mice were previously published by Borgna & Deberle et al., Mol Pharm 2022 [3] (Copyright 2022, American Chemical Society) and were added for comparison.

[^{225}Ac]Ac-SibuDAB, applied at an activity level of 5 kBq or 10 kBq, was slightly more effective than using a 1000-fold higher activity of [^{177}Lu]Lu-SibuDAB (5 MBq or 10 MBq per mouse) (Figure S7A,B). Two out of six tumors of mice treated with 5 MBq and five out of six tumors of mice treated with 10 MBq [^{177}Lu]Lu-SibuDAB completely disappeared without regrowth until Day 56. All other tumors of mice in these groups started to regrow after ~4 and ~7 weeks, respectively. In comparison, no tumors were visible 56 days after injection of 5 kBq [^{225}Ac]Ac-SibuDAB. Application of 5 kBq [^{225}Ac]Ac-PSMA-617 showed approximately the same anti-tumor effect as 10 MBq [^{177}Lu]Lu-PSMA-617 (Figure S7C,D).

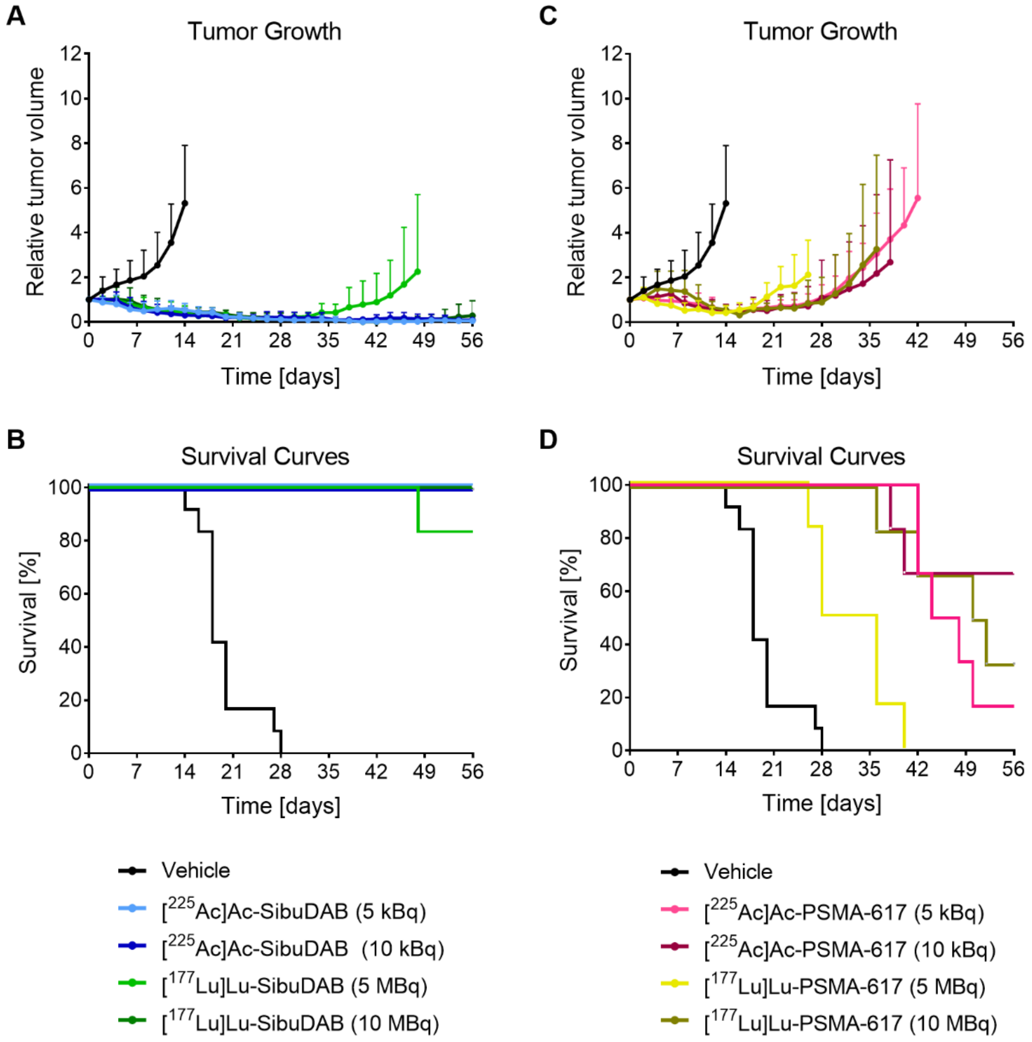

Figure S7. Comparison of the therapeutic efficacy of the radioligands in combination with actinium-225 and lutetium-177. (**A**,**C**) Relative tumor growth curves shown until the first mouse of the respective group reached an endpoint. (**B**,**D**) Kaplan-Meier plots showing the survival curves of mice of each group. Data of mice injected with 5 MBq [^{177}Lu]Lu-SibuDAB were previously published by Borgna & Deberle et al., Mol Pharm. 2022 [3] (Copyright 2022, American Chemical Society) and data of mice treated with 5 MBq or 10 MBq [^{177}Lu]Lu-PSMA-617 were previously published by Umbricht et al., Mol Pharm. 2018 [8] and Tschan et al., Eur J Nucl Med Mol Imaging 2022 [4] (Copyright 2018, American Chemical Society), respectively, and added for comparison.

10. Comparison of [^{225}Ac]Ac-SibuDAB-Treated Mice with Healthy Mice at Study End

Purpose: To investigate whether a successful tumor treatment with [^{225}Ac]Ac-SibuDAB was also well tolerated in these mice, several parameters of cured mice were compared to treatment-naïve, non-tumor-bearing mice on Day 56.

Methods: In order to assess the tolerability of the treatment, the body mass, the organ masses and organ-to-brain mass ratios as well as blood chemistry of the cured mice were compared to those of healthy, non-tumor-bearing BALB/c nude mice on Day 56.

Results: *Survival:* Targeted alpha therapy using [^{225}Ac]Ac-SibuDAB was very effective and all mice treated with 5 kBq, 10 kBq or 30 kBq of this radioligand survived until the end of the study with no tumor visible on Day 56.

Body Mass: The body mass of mice that were effectively treated with [^{225}Ac]Ac-SibuDAB was in the same range as for healthy, non-tumor-bearing control mice at the end of the study on Day 56 (p>0.05) (Table S11).

Table S11. Relative body mass (RBM) and organ masses of therapy mice that survived until the end of the study on Day 56.

Group	Tumor at therapy start	RBM	Kidney mass [mg]	Liver mass [mg]	Spleen mass [mg]	Brain mass [mg]
Control	none	1.14 ± 0.03	286 ± 19	1152 ± 83	129 ± 30	390 ± 7
[225Ac]Ac-SibuDAB (5 kBq)	PC-3 PIP	1.13 ± 0.05	252 ± 15*	1079 ± 47	113 ± 17	403 ± 14
[225Ac]Ac-SibuDAB (10 kBq)	PC-3 PIP	1.12 ± 0.03	260 ± 11	1139 ± 83	116 ± 32	384 ± 18
[225Ac]Ac-SibuDAB (30 kBq)	PC-3 PIP	1.11 ± 0.02	252 ± 24	1096 ± 79	127 ± 23	408 ± 16

* Significantly different from healthy non-tumor-bearing control mice ($p<0.05$).

Organ-to-Brain Mass Ratios: Mice that received 5 kBq or 30 kBq [225Ac]Ac-SibuDAB showed significantly decreased kidney-to-brain mass ratios (0.63 ± 0.03 and 0.62 ± 0.05, respectively) as compared to healthy control mice of the same age (0.73 ± 0.04; $p<0.05$; Table S12). A trend of lower kidney-to-brain mass ratios were also observed for mice treated with 10 kBq [225Ac]Ac-SibuDAB ($p>0.05$). No significant differences were found in the organ mass or organ-to-brain mass ratio of the liver or spleen after the application of up to 30 kBq [225Ac]Ac-SibuDAB (Table S11,S12).

Table S12. Organ-to-brain mass ratios of mice treated with [225Ac]Ac-SibuDAB that survived until the end of the study on Day 56.

Group	Tumor at therapy start	Kidney-to-brain mass ratio	Liver-to-brain mass ratio	Spleen-to-brain mass ratio
Control	none	0.73 ± 0.04	3.0 ± 0.2	0.33 ± 0.08
[225Ac]Ac-SibuDAB (5 kBq)	PC-3 PIP	0.63 ± 0.03*	2.7 ± 0.1	0.28 ± 0.04
[225Ac]Ac-SibuDAB (10 kBq)	PC-3 PIP	0.68 ± 0.04	3.0 ± 0.2	0.31 ± 0.09
[225Ac]Ac-SibuDAB (30 kBq)	PC-3 PIP	0.62 ± 0.05*	2.7 ± 0.2	0.31 ± 0.07

* Significantly different from healthy non-tumor-bearing control mice ($p<0.05$).

Blood Chemistry: Blood plasma analysis of blood urea nitrogen (BUN), alkaline phosphatase (ALP), total bilirubin (TBIL) and albumin (ALB) revealed no differences between treated and untreated BALB/c nude mice ($p>0.05$; Table S13).

Table S13. Blood urea nitrogen (BUN), alkaline phosphatase (ALP), total bilirubin (TBIL) and albumin (ALB) levels of therapy mice that survived until the end of the study on Day 56.

Group	Tumor at therapy start	Surviving mice	BUN [mmol/L]	ALP [U/L]	TBIL [µmol/L]	ALB [g/L]
Control	none	4/4	6.7 ± 1.3	82 ± 6	3.5 ± 1.0	24 ± 2
[225Ac]Ac-SibuDAB (5 kBq)	PC-3 PIP	6/6	6.4 ± 1.2	93 ± 10	4.0 ± 0.9	25 ± 2
[225Ac]Ac-SibuDAB (10 kBq)	PC-3 PIP	6/6	7.3 ± 0.8	85 ± 7	3.2 ± 0.4	26 ± 2
[225Ac]Ac-SibuDAB (30 kBq)	PC-3 PIP	4/4	7.2 ± 0.7	83 ± 2	4.3 ± 0.5	22 ± 1

No values were significantly different from healthy non-tumor-bearing control mice ($p<0.05$).

References

1. Deberle, L.M.; Benešová, M.; Umbricht, C.A.; Borgna, F.; Büchler, M.; Zhernosekov, K.; Schibli, R.; Müller, C. Development of a new class of PSMA radioligands comprising ibuprofen as an albumin-binding entity. *Theranostics* **2020**, *10*, 1678-1693, doi:10.7150/thno.40482.
2. Benešová, M.; Umbricht, C.A.; Schibli, R.; Müller, C. Albumin-binding PSMA ligands: optimization of the tissue distribution profile. *Mol Pharm* **2018**, *15*, 934-946, doi:10.1021/acs.molpharmaceut.7b00877.
3. Borgna, F.; Deberle, L.M.; Busslinger, S.D.; Tschan, V.J.; Walde, L.M.; Becker, A.E.; Schibli, R.; Müller, C. Preclinical investigations to explore the difference between the diastereomers [^{177}Lu]Lu-SibuDAB and [^{177}Lu]Lu-RibuDAB toward prostate cancer therapy. *Mol Pharm* **2022**, doi:10.1021/acs.molpharmaceut.1c00994.
4. Tschan, V.J.; Borgna, F.; Busslinger, S.D.; Stirn, M.; Rodriguez, J.M.M.; Bernhardt, P.; Schibli, R.; Müller, C. Preclinical investigations using [^{177}Lu]Lu-Ibu-DAB-PSMA toward its clinical translation for radioligand therapy of prostate cancer. *Eur J Nucl Med Mol Imaging* **2022**, doi:10.1007/s00259-022-05837-2.
5. Deberle, L.M.; Tschan, V.J.; Borgna, F.; Sozzi-Guo, F.; Bernhardt, P.; Schibli, R.; Müller, C. Albumin-binding PSMA radioligands: impact of minimal structural changes on the tissue distribution profile. *Molecules* **2020**, *25*, doi:10.3390/molecules25112542.
6. Binder, T.; Diem, H.; Fuchs, R.; Gutensohn, K.; Nebe, T. Pappenheim stain: description of a hematological standard stain - history, chemistry, procedure, artifacts and problem solutions. *J Lab Med* **2012**, *36*, 293-309, doi:10.1515/labmed-2012-0027.
7. Schneck, K.; Washington, M.; Holder, D.; Lodge, K.; Motzel, S. Hematologic and serum biochemical reference values in non-transgenic FVB mice. *Comp Med* **2000**, *50*, 32-35.
8. Umbricht, C.A.; Benešová, M.; Schibli, R.; Müller, C. Preclinical development of novel PSMA-targeting radioligands: modulation of albumin-binding properties to improve prostate cancer therapy. *Mol Pharm* **2018**, *15*, 2297-2306, doi:10.1021/acs.molpharmaceut.8b00152.
